# A structured biomimetic nanoparticle as inflammatory factor sponge and autophagy-regulatory agent against intervertebral disc degeneration and discogenic pain

**DOI:** 10.1186/s12951-024-02715-x

**Published:** 2024-08-14

**Authors:** Kanglu Li, Wenbo Yang, Xuanzuo Chen, Yihan Yu, Yiran Liu, Feifei Ni, Yan Xiao, Xiangcheng Qing, Sheng Liu, YuXin He, Baichuan Wang, Li Xu, Zengwu Shao, Lei Zhao, Yizhong Peng, Hui Lin

**Affiliations:** 1 Department of Orthopaedics, Union Hospital, Tongji Medical College, Huazhong University of Science and Technology, Wuhan, 430022 China; 2 Department of Radiology, Union Hospital, Tongji Medical College, Huazhong University of Science and Technology, Wuhan, 430022 China; 3 Department of Emergency, Union Hospital, Tongji Medical College, HuaZhong University of Science and Technology, Wuhan, 430022 China; 4 Tongji Medical College, HuaZhong University of Science and Technology, Wuhan, 430030 China

**Keywords:** Intervertebral disc degeneration, Targeting nanoparticle, Biomimetic, Discogenic pain, Innervation

## Abstract

Lower back pain (LBP) is a common condition closely associated with intervertebral disc degeneration (IDD), causing a significant socioeconomic burden. Inflammatory activation in degenerated discs involves pro-inflammatory cytokines, dysregulated regulatory cytokines, and increased levels of nerve growth factor (NGF), leading to further intervertebral disc destruction and pain sensitization. Macrophage polarization is closely related to autophagy. Based on these pathological features, a structured biomimetic nanoparticle coated with TrkA-overexpressing macrophage membranes (TMNP@SR) with a rapamycin-loaded mesoporous silica core is developed. TMNP@SR acted like sponges to adsorbe inflammatory cytokines and NGF and delivers the autophagy regulator rapamycin (RAPA) into macrophages through homologous targeting effects of the outer engineered cell membrane. By regulating autophagy activation, TMNP@SR promoted the M1-to-M2 switch of macrophages to avoid continuous activation of inflammation within the degenerated disc, which prevented the apoptosis of nucleus pulposus cells. In addition, TMNP@SR relieved mechanical and thermal hyperalgesia, reduced calcitonin gene-related peptide (CGRP) and substance P (SP) expression in the dorsal root ganglion, and downregulated GFAP and c-FOS signaling in the spinal cord in the rat IDD model. In summary, TMNP@SR spontaneously inhibits the aggravation of disc inflammation to alleviate disc degeneration and reduce the ingress of sensory nerves, presenting a promising treatment strategy for LBP induced by disc degeneration.

**Graphical Abstract:**

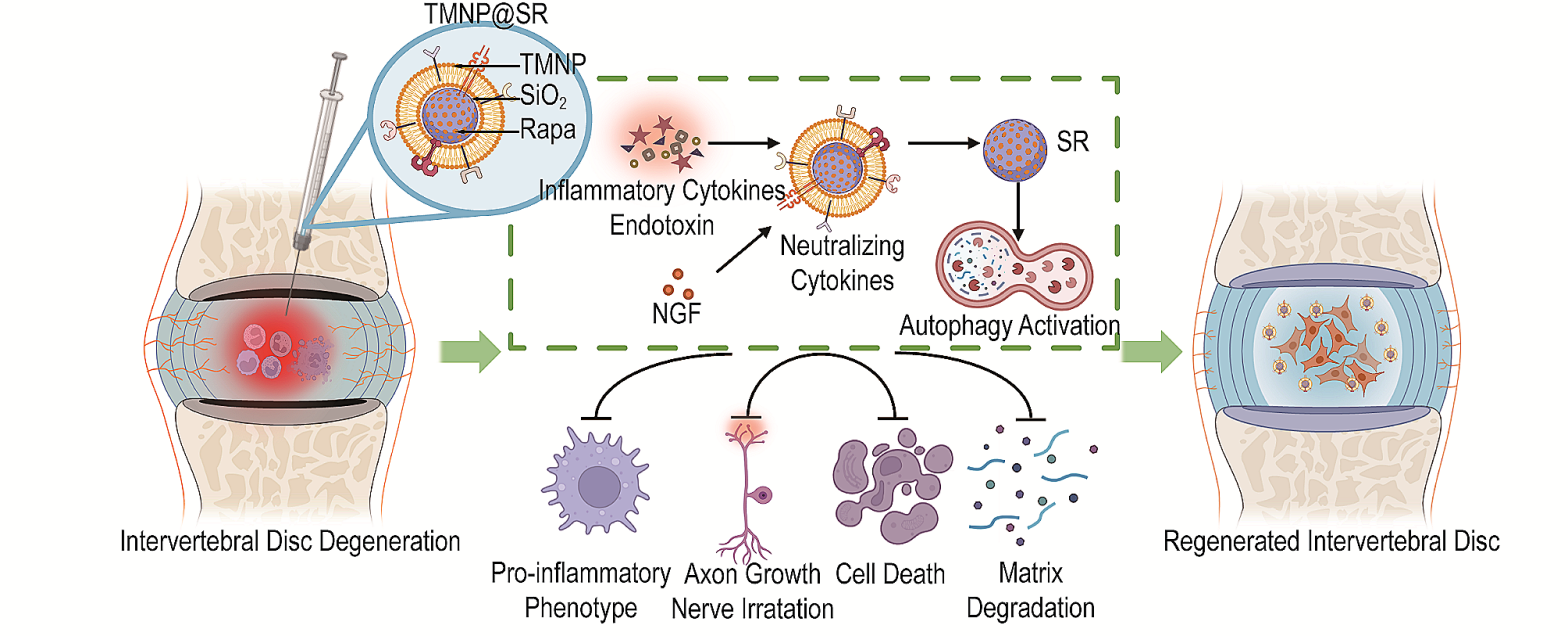

**Supplementary Information:**

The online version contains supplementary material available at 10.1186/s12951-024-02715-x.

## Introduction

Low back pain (LBP) is a global healthcare concern that causes productivity loss worldwide and is the leading cause of years lived with disability in 126 countries [1]. Intervertebral disc (IVD) degeneration (IDD), the basic pathological process of spinal degeneration, is the leading cause of LBP and is observed in nearly 40% of patients with LBP [2, 3]. Traditional treatments for IDD, including physiotherapy, nonsteroidal anti-inflammatory drug administration, and surgery, mostly treat painful symptoms but fail to intervene in the pathological etiology of IDD development. These approaches have limited therapeutic effects and potential adverse effects due to prolonged drug administration, as well as the incidence of reherniation and adjacent disc degeneration [2, 3].

The immune balance in the microenvironment is the primary cause of IDD. Studies have shown that chronic inflammatory responses are closely associated with the establishment and progression of IDD [4, 5]. The chronic inflammatory microenvironment is established by disc cells with a pro-inflammatory phenotype and infiltrating inflammatory cells with increased secretion of pro-inflammatory mediators [6], among which M1 macrophage-mediated inflammatory responses play a crucial role in IDD [7]. M1 macrophages release various cytokines and promote the secretion of pro-inflammatory cytokines and chemokines from disc cells, leading to an immune response cascade [8]. Moreover, secreted cytokines induce cell apoptosis and senescence and increase the release of proteolytic enzymes, leading to impaired endogenous regeneration and extracellular matrix (ECM) breakdown [9–11].

The accumulated inflammatory mediators in degenerated discs, including TNF-α, IL-1β, IL-6 and interferon (IFN)-γ, also inflame the sensory endings from dorsal root ganglion (DRG) neurons and activate the algic signal transmitting pathway by regulating the activity of ion channels and the production of sensory neuropeptides, such as SP and CGRP [12–15]. Increased expression of TNF-a, IL-1β, IL-6, and IL-8 has been identified in the spinal cord and DRGs in animal models of LBP, and it maintains the hypersensitive state of sensory nerves [16, 17]. Nerve ingrowth into the IVD, mediated by nerve growth factor (NGF), which belongs to the family of neurotrophins, facilitates sensitization of afferent nerves by the inflammatory microenvironment [18]. Upon binding to tropomyosin receptor kinase A (TrkA), NGF mediates a broad spectrum of neuronal biological behaviors, including survival, proliferation, differentiation, and chemotaxis [19]. Inflammatory mediators stimulate NGF expression in disc cells [20, 21]. Therefore, regulating the local inflammatory microenvironment is of considerable interest not only to enhance the regeneration of damaged tissue but also to regulate nerve ingrowth and neuroinflammation in degenerated discs.

Nanotechnology and materials science based on engineered liposomes, cell membrane nanosponges, and exosomes have shown great potential for binding and neutralizing bacterial toxins [22–24]. Biomimetic synthetic strategies that harvest cellular membranes with natural proteins enable the efficient development of a biomimetic nanomedicine platform that displays proteins with their native orientation, structure, and activity as decoys for biodetoxification applications [25–27]. Macrophages naturally bind to various cytokines and endotoxins with specific receptors on their surface to activate the corresponding signaling pathway [28, 29]. Genetic modification is a feasible strategy for overexpressing certain proteins on the cell surface to functionalize the cell membrane [30].

**Fig. 1:**
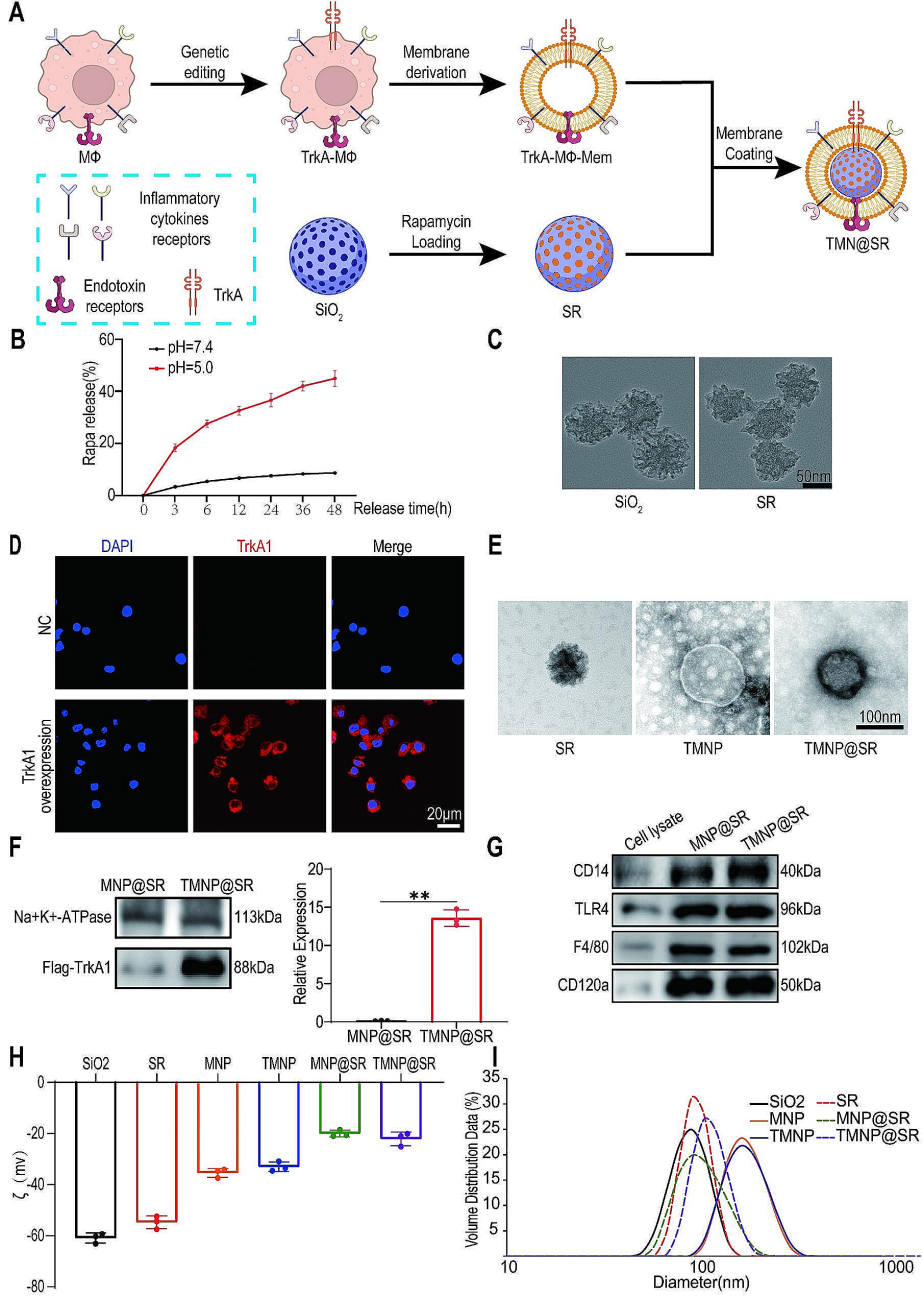
Formulation and characterization of TrkA overexpressed macrophage-like SiO_2_-RAPA nanoparticles (TMNP@SR). (**A**) Schematic representation of TMNP@SR preparation. (**B**) Accumulative release profile of RAPA in SR nanoparticles in the solution with a pH 5 or 7.4 (biological replicates, Data are presented as the mean ± SD, *n* = 3). (**C**) Scanning electron microscopy images of the prepared nanoparticles SiO_2_ and SR. Scale bar: 50 nm. (**D**) Fluorescent images of macrophages with NC and TrkA expression. Scale bars: 20 μm. (**E**) Transmission electron microscopy of SR, TMNP, and TMNP@SR, negatively stained with phosphotungstic acid. Scale bar: 100 nm. (**F**) Representative western blotting plots and statistical analysis of MNP@SR and TMNP@SR (biological replicates, Data are presented as the mean ± SD, *n* = 3). (**G**) Representative protein bands of macrophage cell lysate, MNP@SR, and TMNP@SR, resolved using western blotting. (**H**) Surface zeta potential (ζ, millivolts) of SiO_2_, SR, MNP, TMNP, MNP@SR, and TMNP@SR measured by dynamic light scattering (biological replicates, Data are presented as the mean ± SD, *n* = 3). (**I**) Hydrodynamic size (diameter, nanometers) of SiO_2_, SR, MNP, TMNP, MNP@SR, and TMNP@SR measured by dynamic light scattering. ns, non-significant, *p < 0.05, **p < 0.01, ***p < 0.001. SiO_2_, silica nanoparticles; SR, mesoporous silica nanoparticles loaded with rapamycin; MNP, macrophage-like nanoparticles; MNP@SR, macrophage-like SiO_2_-RAPA nanoparticles; TMNP@SR, TrkA overexpressed macrophage-like SiO_2_-RAPA nanoparticles

Therefore, a cellular nanovesicle consisting of a cellular membrane derived from macrophages overexpressing the NGF receptor TrkA was developed, which was expected to effectively bind to various inflammatory mediators and NGF. The nanovesicles were denoted as TrkA-overexpressing macrophage-like nanoparticles (TMNP). Moreover, cellular membranes are promising materials for nanocores [31]. TMNP were used to encapsulate the mesoporous silica loaded with rapamycin (SR), which was denoted as TMNP@SR. Rapamycin induces autophagy, which mediates the M1 to M2 transformation of macrophages [32]. In this design, the established biomimetic nanodecoys inherit abundant TrkA and other receptors from macrophages to neutralize NGF and inflammatory cytokines and can regulate autophagy to further modify the inflammatory microenvironment, thus effectively achieving attenuation of intervertebral disc degeneration and relieving discogenic pain (Fig. 1).

## Results

### Preparation and characterization of TMNP@SR

**Fig. 2:**
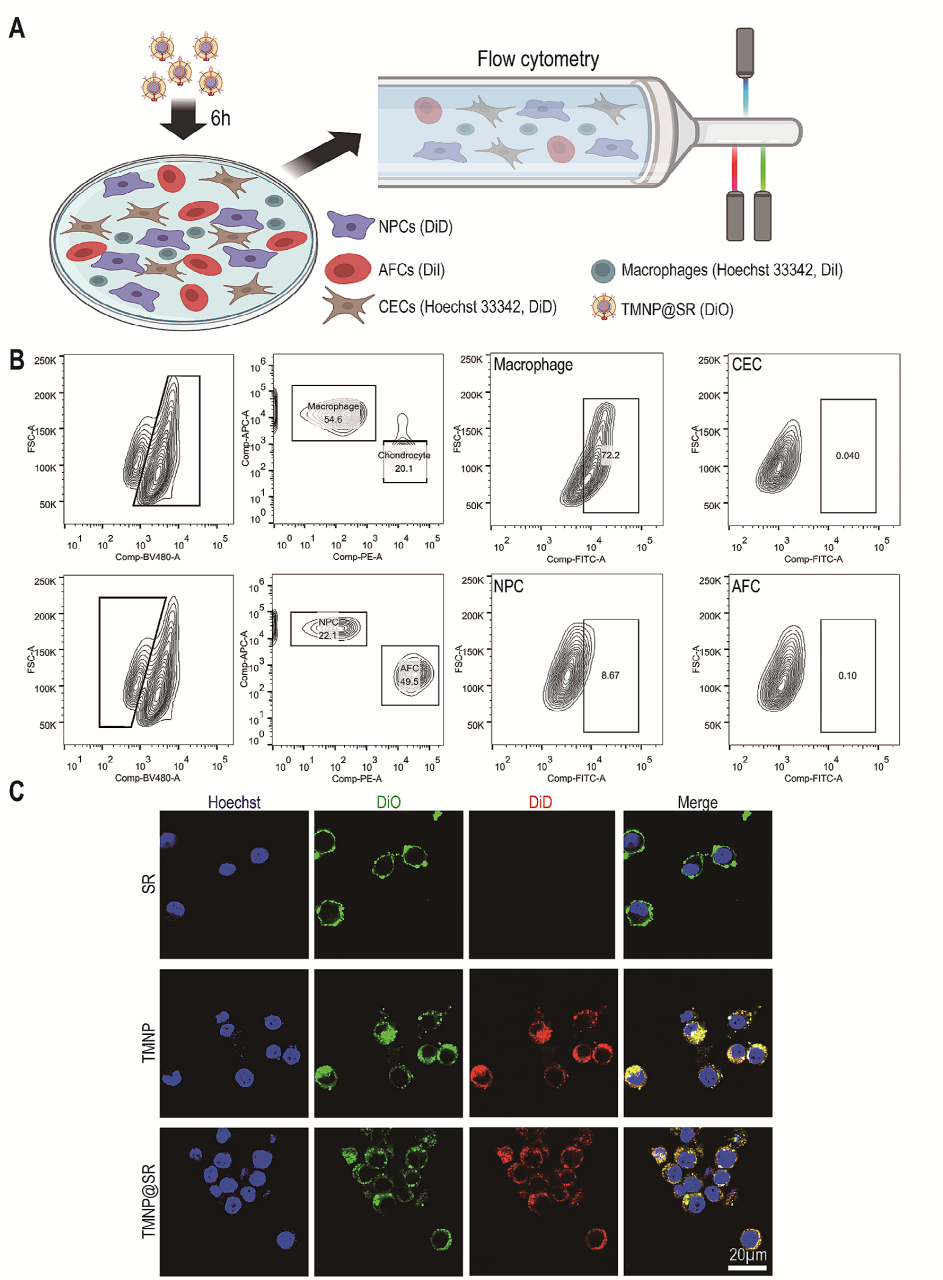
TMNP@SR specifically targeted macrophages in vitro. (**A**) Schematic representation of flow cytometry analysis of the targeting effects of TMNP@SR. (**B**) Flow cytometry evidence of nanoparticle uptake by different cells. (**C**) Fluorescence images showing the uptake of nanoparticles by macrophages. Macrophages were stained with DIO, whereas TMNP or TMNP@SR were stained with DID. Scale bars: 20 μm. SR, mesoporous silica nanoparticles loaded with rapamycin; TMNP, TrkA overexpressed macrophage-like nanoparticles; TMNP@SR, TrkA overexpressed macrophage-like SiO_2_-RAPA nanoparticles; NPCs, nucleus pulposus cells; AFCs, annulus fibrosus cells; CECs, endplate cells

The core of TMNP@SR consists of mesoporous SiO_2_ nanoparticles loaded with RAPA, which are encapsulated with the membrane derived from TrkA-overexpressing macrophages (TrkA-MΦ) (Fig. 1A). The solubility of RAPA in water is relatively limited, with only 2.6 µg/mL at 25 °C [33]. This led to limitations in the use of RAPA alone. In this study, the drug loading capacity of SiO_2_ loaded with RAPA was 42.5%. Considering the lower intracellular pH [34, 35], the SR drug release curve was evaluated to confirm its sustained release in cells. Specifically, in acidic environments (pH 5), SR exhibited significantly higher release rates than at neutral pH (pH 7.5) (Fig. 1B). These studies confirmed the efficient drug-loading capacity of SR and its selective sustained release ability in cells. As shown in Fig. 1C, the morphologies of SiO_2_ and SR were almost identical under scanning electron microscope (SEM), both of which had irregular spherical shapes of approximately 90 nm. The outer shell of TMNP@SR was a macrophage membrane that overexpressed TrkA1. Before extracting the cell membrane, the macrophages were transfected with a Lentivirus-mediated over-expression vector of TrkA1. The comparison of the negative control (NC) group and TrkA1 overexpression group demonstrated that the macrophages successfully overexpressed TrkA1 (Fig. 1D). The PCR findings validated this result (Figure S11A). The cell membranes of macrophages in the NC and TrkA1 overexpression groups were denoted as MNP and TMNP, respectively. MNP@SR and TMNP@SR were successfully synthesized using a HandExtractor to encapsulate the SR in the membranes. Under transmission electron microscope (TEM), the shape of the SR was irregularly spherical, whereas TMNP had a vesicular-like membrane structure (Fig. 1E). Next, we measured the encapsulation ratios of the nanomaterials. The membrane was labeled with DiO (green). SiO_2_ and SR were labeled with CY3 (red). The encapsulation ratio was determined by calculating the ratio of the number of particles showing green and red fluorescence to the total number of red fluorescent particles. The experimental results showed that the encapsulation ratio of MNP@SiO_2_, TMNP@SiO_2_, MNP@SR, and TMNP@SR was 92.35%, 92.41%, 92.88%, and 93.42%, respectively (Figure S1B). There was little difference between the groups. TMNP@SR had the structure of an SR core coated with a cell membrane. We also confirmed that the TrkA1 expression was significantly higher in the TMNP@SR group than in the MNP@SR group (Fig. 1F). In addition, western blotting revealed that the typical membrane markers of macrophages in TMNP@SR and MNP@SR, such as CD14, TRL4, F4/80, and CD120a, were positive compared with those in the cell lysate. Coomassie blue staining confirmed the significant differences in protein distribution between cells and nanoparticles (Fig. 2G). Zeta potential and particle size measurements showed that the zeta potentials of SiO_2_, SR, MNP, TMNP, MNP@SR, and TMNP@SR were approximately -60.9 mV, -54.7 mV, -35.4 mV, -33.0 mV, -20.0 mV, and -22.1 mV, respectively, with average sizes of approximately 87.2 nm, 93.3 nm, 176.3 nm, 177.3 nm, 105.5 nm, and 115.6 nm, respectively (Fig. 1H-I).

### Biocompatibility assessment of the nanoparticles

First, the toxicity of RAPA was assessed in various cell types using the CCK-8 assay. The findings revealed that lower concentrations of RAPA (20, 40, and 80 µg/mL) did not induce a notable reduction in cell viability. However, as the RAPA concentration reached 160 µg/mL, cell viability declined, with the most substantial effect observed on macrophage viability (Figure S2A). Subsequently, the toxicity of MNP@SR and TMNP@SR was evaluated. The indicated concentration of RAPA in the nanomaterials was based on the drug-loading capacity of RAPA onto SiO_2_ and the encapsulation ratio of the drug-loaded nanoparticles into MNP or TMNP. The findings revealed that, at low concentrations, neither MNP@SR nor TMNP@SR significantly reduced nucleus pulposus cells (NPCs) viability. However, when the concentration reached 160 µg/mL, the nanoparticles started to decrease NPCs viability (Figure S2B). Based on these results, RAPA concentration of 80 µg/mL was selected. As shown in Figure S2C, macrophages treated with RAPA, SR, and TMNP@SR (80 ug/mL) for 24 h showed no significant change in cell proliferation. In addition, live/dead cell experiments further confirmed these results, in which the macrophage death rate was comparable among the RAPA, SR, and TMNP@SR groups (Figure S2D). Hemolysis tests showed that different concentrations of TMNP@SR (20, 40, 80, 600, 1200, and 2400 µg/mL) did not cause evident hemolysis (hemolysis rate < 0.2) (Figure S2E). TMNP@SR with different RAPA concentrations (20, 40, and 80 µg/mL) was injected into the tail vein of rats. After 30 days, the hears, liver, spleen, lung, and kidney were collected for H&E staining. As shown in Figure S2F, key organs of the rats were not damaged. These results confirmed that TMNP@SR exhibited excellent biocompatibility.

### NGF-, LPS-, and inflammatory factor-capturing ability of TMNP@SR

Macrophage membranes contain various receptors that allow TMNP@SR to absorb cytokines and modulate the inflammatory microenvironment (Figure S3A). TLR4 binds to lipopolysaccharides(LPS) with the help of LPS-binding protein (LBP), CD14, and MD-2, which are present on the surface of macrophages [36]. Therefore, the ability of TMNP@SR to remove LPS was quantified using two sets of experiments. Firstly, the concentration of nanomaterials was controlled at 80 µg/mL, and they were incubated with different concentrations of LPS (8.0, 4.0, 2.0, and 1.0 µg/mL) at 37 °C for 1 h. The supernatant was collected after ultracentrifugation to evaluate the residual LPS. When LPS decreased to 2.0 µg/mL, the residual LPS concentration in the supernatant of MNP@SR and TMNP@SR reached its lowest point (Figure S3B). Then, the concentration of LPS was controlled at 4.0 µg/mL, and the concentrations of nanomaterials differed (0, 10, 20, 40, and 80 µg/mL). When the concentrations of MNP@SR and TMNP@SR increased from 0 µg/mL to 80 µg/mL, a linear decrease in residual LPS was detected in the supernatant (Figure S3C). Similarly, the ability of nanomaterials to sequester proinflammatory cytokines, including TNF-α, IFN-γ, IL-1β, and IL-6, was also evaluated. After incubation at 37 °C for 1 h, the nanoparticles were removed by ultracentrifugation, and the residual cytokines in the supernatant were determined using ELISA. As shown in Figure S3D-G, when the concentration of MNP@SR and TMNP@SR reached 80 µg/mL, the removal rate of cytokines by nanoparticles reached its maximum, namely 53.13% and 53.14% for TNF-α, 61.03% and 55.09% for IFN-γ, 59.72% and 59.96% for IL-1β, and 58.74% and 67.72% for IL-6. Next, the ability of TMNP@SR to bind to NGF was investigated. Nanoparticles (MNP@SR and TMNP@SR) (20, 40, and 80 µg/mL) were mixed with NGF (1000 pg/mL). Compared to MNP@SR, TMNP@SR exhibited higher NGF-scavenging efficiency, particularly when the concentration of nanomaterials reached 80 µg/mL, indicating improved NGF-binding capacity after overexpressing TrkA1 (Figure S3H). Moreover, MNP@SR had a certain ability to capture NGF, which might be attributed to the native expression of TrkA1 receptors on macrophages [37]. The above results validated the ability of TMNP@SR to capture NGF, LPS, and inflammatory factors and indicate its potential applications in relieving inflammation and pain.

### TMNP@SR can be specifically engulfed by macrophages

The targeted effects of nanomaterials on specific cells ensure high efficacy and low toxicity. The homology of cell membranes [38] and phagocytic capacity of macrophages for exogenous substances [39] might contribute to the specific uptake of TMNP@SR by macrophages. TMNP@SR were verified to specifically target and engulfed by macrophages. As shown in Fig. 2A, NPCs were stained with DiD, annulus fibrosus cells (AFCs) with DiI, macrophages with Hoechst 33342 and DiI, cartilage endplate cells (CECs) with Hoechst 33,342 and DiD, and TMNP@SR with DiO. The cells were mixed and treated with TMNP@SR for 6 h. The different cells were differentiated using fluorescence, and the positive rate of DiO (TMNP@SR signals) in the cells was recorded. The experimental results showed that the positivity rate of DiO was 73.2% for macrophages, 8.67% for NPCs, 0.04% for CECs, and 0.7% for AFCs (Fig. 2B). This means that, compared to other cells, macrophages were more likely to take up TMNP@SR. Next, the macrophages were stained with DiO and TMNP or TMNP@SR with DiD and treated with SR, TMNP, or TMNP@SR for 6 h. Laser scanning confocal microscopy showed co-staining of DiO and DiD in macrophages in both TMNP and TMNP@SR groups (Fig. 2C). This indicated that TMNP@SR can be engulfed by macrophages.

### TMNP@SR inhibited macrophage M1 polarization by modulating the inflammatory microenvironment

**Fig. 3:**
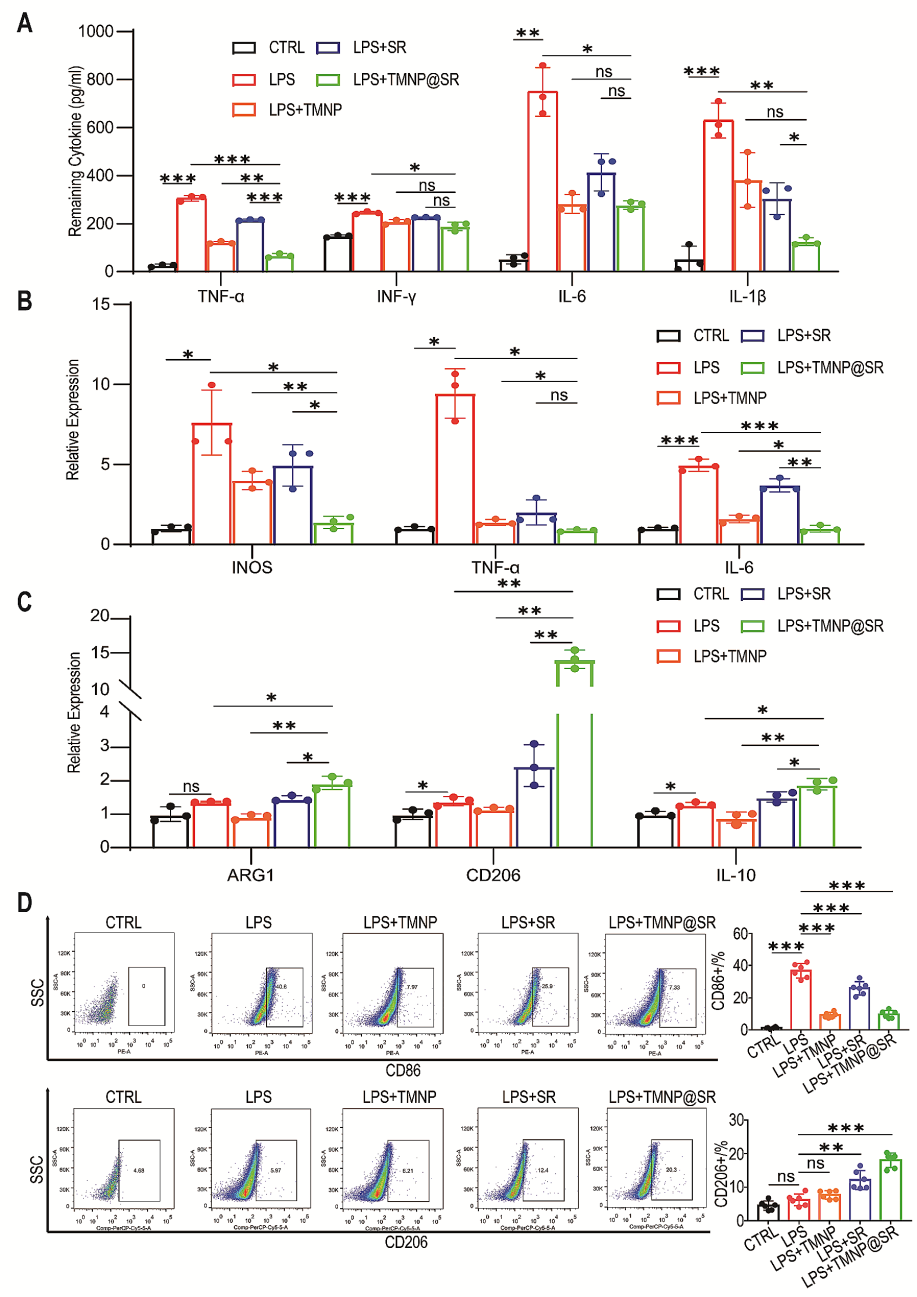
TMNP@SR alleviated the inflammatory environment and inhibited macrophage polarization. (**A**) ELISA assay was used to detect the concentration of inflammatory factors after macrophage treatment with LPS and SR, LPS + TMNP, or LPS + TMNP@SR (biological replicates, Data are presented as the mean ± SD, *n* = 3). Relative mRNA expression of the M1 markers (iNOS, TNF-α, and IL-6) (**B**) and M2 markers (ARG1, CD206, and IL-10) (**C**) were determined by quantitative RT-PCR analysis (biological replicates, Data are presented as the mean ± SD, *n* = 3). (**D**) The typical surface markers CD86 and CD206 of polarized macrophages were determined by flow cytometry (biological replicates, Data are presented as the mean ± SD, *n* = 6). ns, non-significant, *p < 0.05, **p < 0.01, ***p < 0.001. SR, mesoporous silica nanoparticles loaded with rapamycin; TMNP, TrkA overexpressed macrophage-like nanoparticles; TMNP@SR, TrkA overexpressed macrophage-like SiO_2_-RAPA nanoparticles

M1 polarization of macrophages plays a critical role in the degenerative intervertebral disc environment by releasing inflammatory factors that further stimulate M1 polarization of macrophages. Using LPS (2 µg/mL), macrophage polarization was stimulated to simulate a degenerative environment. Simultaneously, nanomaterials were added to the culture medium for 6 h. The cell supernatants were extracted to detect inflammatory cytokines. The inflammatory factors in the LPS group increased significantly compared to the CTRL (PBS only) group, including TNF-α, IFN-γ, IL-1β, and IL-6 (Fig. 3A). When both TMNP@SR and LPS were added, the inflammatory factors in the supernatant were significantly downregulated compared with those in the LPS group. Compared with the LPS + SR group, cytokines in LPS + TMNP@SR group were relatively lower, particularly TNF-α and IL-1β. The macrophage polarization phenotype was also analyzed. M1 polarization markers including iNOS, TNF-α, and IL-6 were upregulated in the LPS group, whereas TMNP and TMNP@SR decreased the expression levels of these markers; this effect was more pronounced for TMNP@SR (Fig. 3B). In addition, the relative mRNA expression levels of M2 polarization markers, including ARG1, CD206, and IL-10, were tested under the same conditions. The results showed that LPS slightly increased the expression of M2 polarization markers, whereas TMNP diminished this effect (Fig. 3C). In contrast, the expression levels of ARG1, CD206, and IL-10 were increased in the LPS + SR and LPS + TMNP@SR groups, indicating the promoting effects of SR on macrophage M2 polarization. The flow cytometry results further confirmed that TMNP@SR effectively alleviated LPS-induced M1 polarization of macrophages. Compared to the LPS group (36.75 ± 4.52%), the proportion of CD86^+^ macrophages in the LPS + TMNP (9.18 ± 1.73%), LPS + SR (25.9 ± 4.15%), and LPS + TMNP@SR groups (9.68 ± 2.38%) was significantly lower (Fig. 3D). Moreover, the proportions of CD206 macrophages in the LPS + TMNP, LPS + SR, and LPS + TMNP@SR groups were compared, and CD206 was approximately 7.7 ± 1.26%, 12.16 ± 2.80%, and 18.02 ± 2.17%. This indicated that both the TMNP outer membrane and SR inner core inhibited macrophage M1 polarization, whereas SR promoted M2 polarization, achieving an M1-to-M2 switch. In addition, the comparison of the LPS + SR and LPS + TMNP@SR groups showed an improvement in the modulatory effects of SR on macrophage polarization phenotypes.

### TMNP@SR promoted macrophage M2 polarization by autophagy activation in vitro

**Fig. 4:**
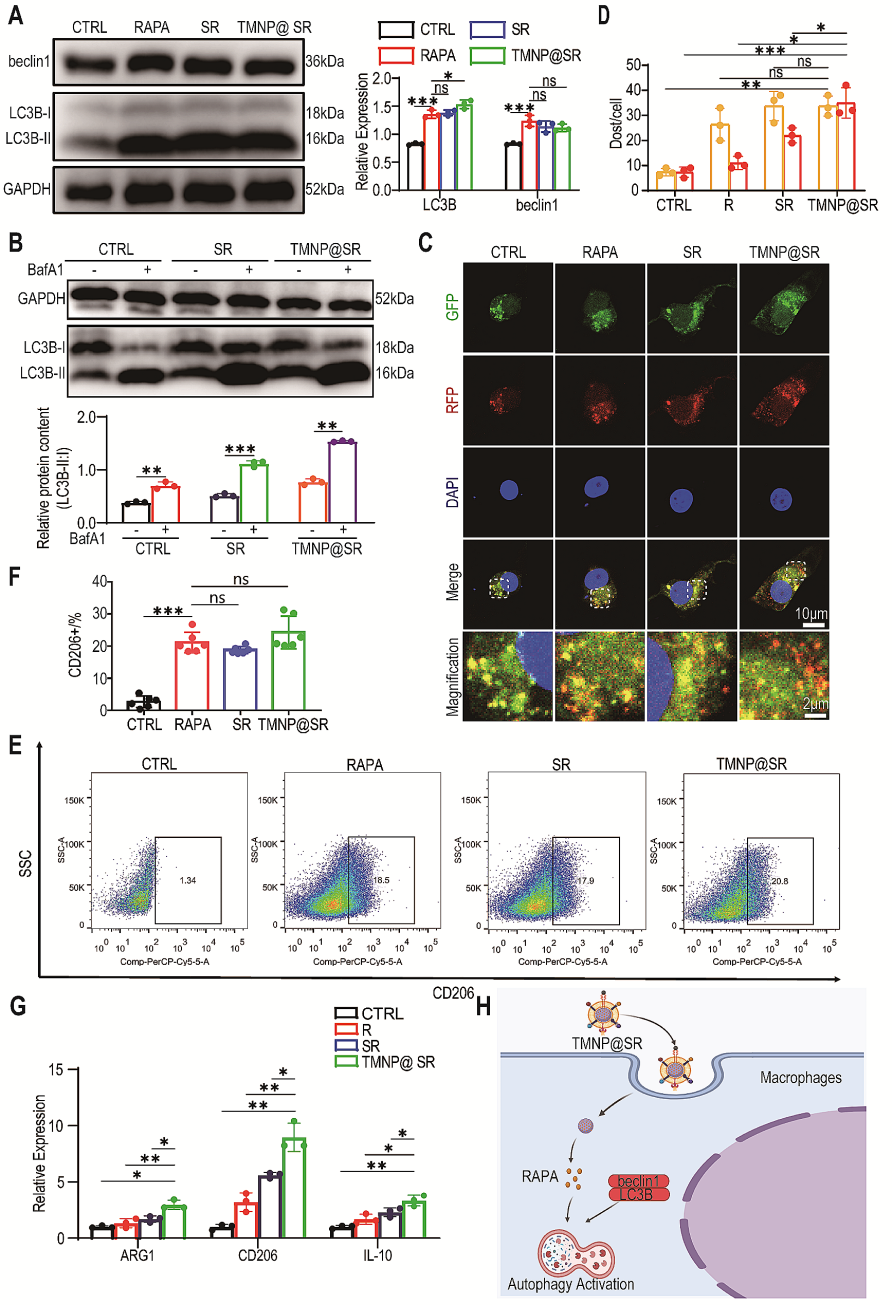
TMNP@SR promoted the polarization of M2 macrophages by activating autophagy. (**A**) Representative western blotting plots and statistical analysis of LC3B and beclin1 in macrophages treated with RAPA, SR, and TMNP@SR (biological replicates, Data are presented as the mean ± SD, *n* = 3). (**B**) Western blot analysis of LC3B-II: I in macrophages treated with RAPA, SR, and TMNP@SR. BafA1 was added in the last 4 h (biological replicates, Data are presented as the mean ± SD, *n* = 3). (**C**) Representative immunofluorescence images of macrophages expressing mRFP-GFP-LC3. Nuclei are blue (DAPI). Scale bars: 10 μm. Magnification scale bars: 2 μm. (**D**) Statistical analysis of autophagosomes (yellow dots, GFP + RFP+) and autolysosomes (red dots, GFP-RFP+) in macrophages treated with RAPA, SR, or TMNP@SR (biological replicates, Data are presented as the mean ± SD, *n* = 3) (**E**) The typical surface marker, CD206, of polarized macrophages was determined using flow cytometry (**F**) Statistical analysis of the percentage of CD206 + cells in different groups (biological replicates, Data are presented as the mean ± SD, *n* = 6). (**G**) Relative mRNA expression of the M2 markers, ARG1, CD206, and IL-10 was determined in macrophages treated with RAPA, SR, or TMNP@SR (biological replicates, Data are presented as the mean ± SD, *n* = 3). (**H**) Schematic representation of TMNP@SR delivering RAPA into macrophages to effectively promote autophagy. ns, non-significant, *p < 0.05, **p < 0.01, ***p < 0.001. R, rapamycin; SR, mesoporous silica nanoparticles loaded with rapamycin; TMNP@SR, TrkA overexpressed macrophage-like SiO_2_-RAPA nanoparticles

Next, the mechanisms by which TMNP@SR promoted M2 polarization were analyzed. After treating the macrophages with RAPA (80 µg/mL), SR (80 ug/mL), and TMNP@SR (80 ug/mL) for 12 h, the relative expression levels of LC3B and Beclin1 were increased, indicating the activation of macrophage autophagy (Fig. 4A). Bafilomycin A 1 (BafA1), which prevents the fusion of autophagosomes and lysosomes, was used to analyze autophagy. After the addition of BafA1, the LC3B-II levels of RAPA, SR, and TMNP@SR increased, whereas those of LC3B-I decreased (Fig. 4B). To evaluate the relative abundance of autophagosomes and autolysosomes after the nanomaterials acted on macrophages, adenovirus-transduced mCherry-GFP tandem-tagged LC3 was used (Fig. 4C). The yellow dots and red dots represent autophagosomes and autolysosomes, respectively. After treating macrophages with RAPA, SR, or TMNP@SR, the abundance of autophagosomes significantly increased, and no significant difference between the groups was observed (Fig. 4D). The number of autolysosomes in the TMNP@SR group was significantly higher than that in the other groups. These results indicated that TMNP@SR stimulated autophagy by enhancing the autophagic flux. Next, macrophage M2 polarization was observed by detecting the number of CD206^+^ macrophages using flow cytometry (Fig. 4E). The CD206^+^ macrophage ratios of RAPA, SR, and TMNP@SR were 21.12 ± 3.18%, 18.78 ± 1.11%, and 24.28 ± 5.11%, respectively, all significantly higher than those of CTRL (2.70 ± 1.78%) (Fig. 4F). Relative mRNA expression levels of ARG1, CD206, and IL-10 confirmed these findings (Fig. 4G). As shown in Fig. 4H, TMNP@SR was more easily taken up by macrophages to induce classical autophagy and stimulate macrophage M2 polarization.

### TMNP@SR maintained NPCs viability and regulated extracellular matrix metabolism by interfering with macrophages

**Fig. 5:**
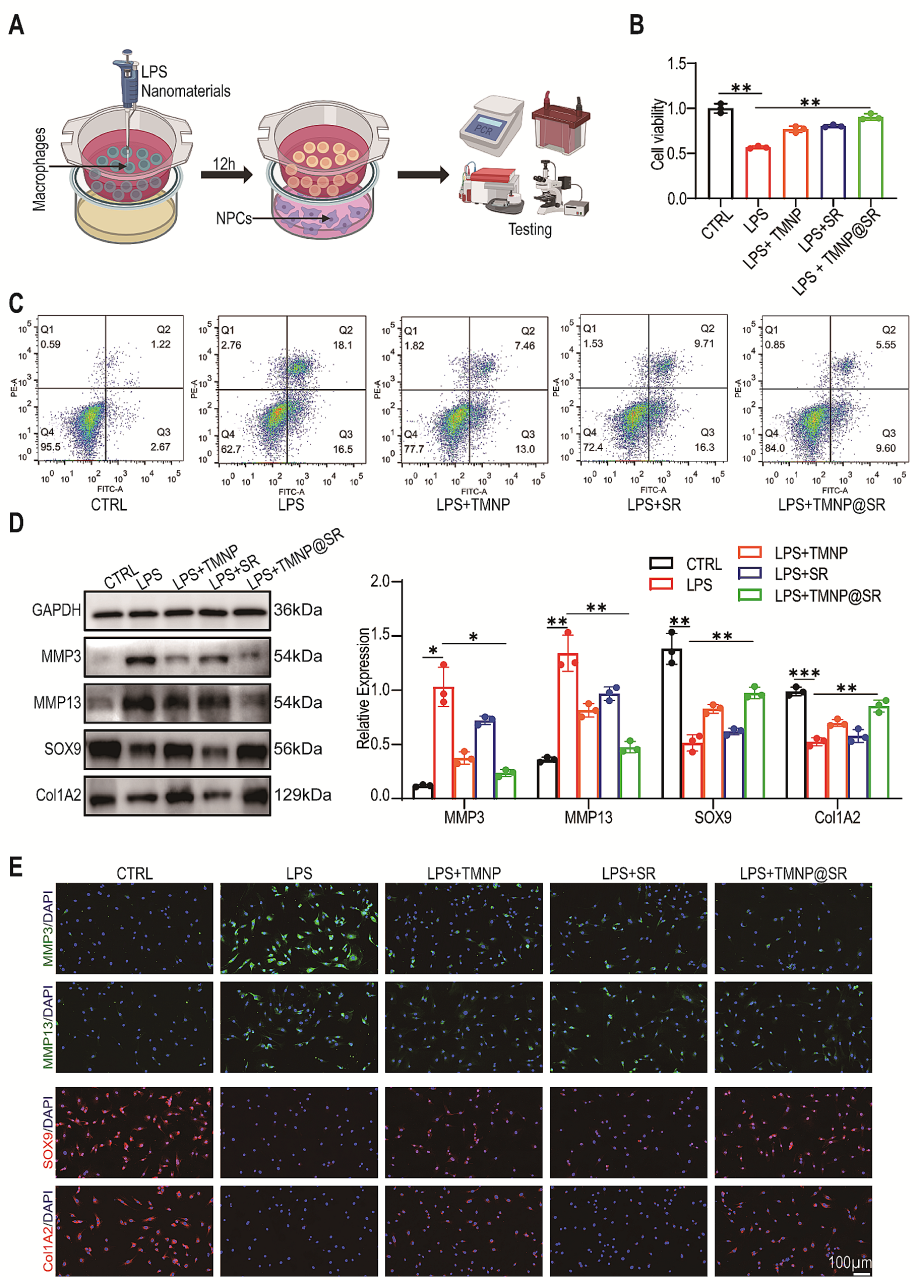
TMNP@SR blocked the effects of M1 polarized macrophages on NPCs death and impaired matrix metabolism. (**A**) Schematic diagram of a co-culture system for simulating the pro-inflammatory microenvironment for NPCs, with M1 polarized macrophages in the upper chamber and NPCs in the lower chamber. (**B**) The cell viability of NPCs with macrophages treated with CTRL, LPS, LPS + SR, LPS + TMNP, and LPS + TMNP@SR (biological replicates, Data are presented as the mean ± SD, *n* = 3). (**C**) Flow cytometry of Annexin V/PI-stained NPCs. (**D**) Representative western blotting plots and statistical analysis of MMP3, MMP13, SOX9, and Col2A1 expression in NPCs (biological replicates, Data are presented as the mean ± SD, *n* = 3). (**E**) Representative immunofluorescence images of MMP3, MMP13, SOX9, and Col2A1 in NPCs with macrophages treated with CTRL, LPS, LPS + SR, LPS + TMNP, and LPS + TMNP@SR. Scale bars: 100 μm. ns, non-significant, *p < 0.05, **p < 0.01, ***p < 0.001. SR, mesoporous silica nanoparticles loaded with rapamycin; TMNP, TrkA overexpressed macrophage-like nanoparticles; TMNP@SR, TrkA overexpressed macrophage-like SiO_2_-RAPA nanoparticles

M1 macrophages mediate a pro-inflammatory microenvironment in IDD, impair extracellular matrix metabolism, and cause cell death [40–42]. As shown in Fig. 5A, we used transwells to establish a co-culture system with macrophages in the upper layer and NPCs in the lower layer to evaluate the protective effects of TMNP@SR on NPCs against the pro-inflammatory microenvironment. First, the macrophages were treated with LPS and nanomaterials for 12 h, followed by co-culture of the upper layer of macrophages with the lower layer of NPCs for 24 h. LPS induced macrophage M1 polarization to simulate the inflammatory activation state of the IVD. Next, nanomaterials (TMNP, SR, and TMNP@SR) were used in a co-culture system, and the viability of NPCs and extracellular matrix metabolism were evaluated. After treating the macrophages with LPS, the viability of the co-cultured NPCs was significantly impaired, which was reversed by TMNP@SR (Fig. 5B). Flow cytometry analysis of apoptosis further confirmed these results. The apoptotic rate in the LPS group was significantly upregulated (36.84 ± 4.55%), whereas TMNP@SR inhibited the LPS-induced cell apoptosis (13.07 ± 4.18%) (Fig. 5C, Figure S4A). Western blot analysis showed that M1 polarized macrophages promoted the expression of NPCs catabolic proteins (MMP3 and MMP13) and downregulated the expression of NPCs synthetic metabolic proteins (COL2A1 and SOX9), indicating matrix degradation (Fig. 5D). After macrophage treatment with TMNP@SR, significantly improved NPC matrix deposition was observed via an increase in the expression of COL2A1 and SOX9 and inhibition of MMP3 and MMP13. These results were verified by immunofluorescence staining (Fig. 5E, Figure S4B). These results demonstrated the beneficial effects of TMNP@SR in modulating the pro-inflammatory microenvironment, leading to improved NPC survival and extracellular matrx metabolism.

### TMNP@SR inhibited DRG growth and pain sensitivity by capturing NGF

**Fig. 6:**
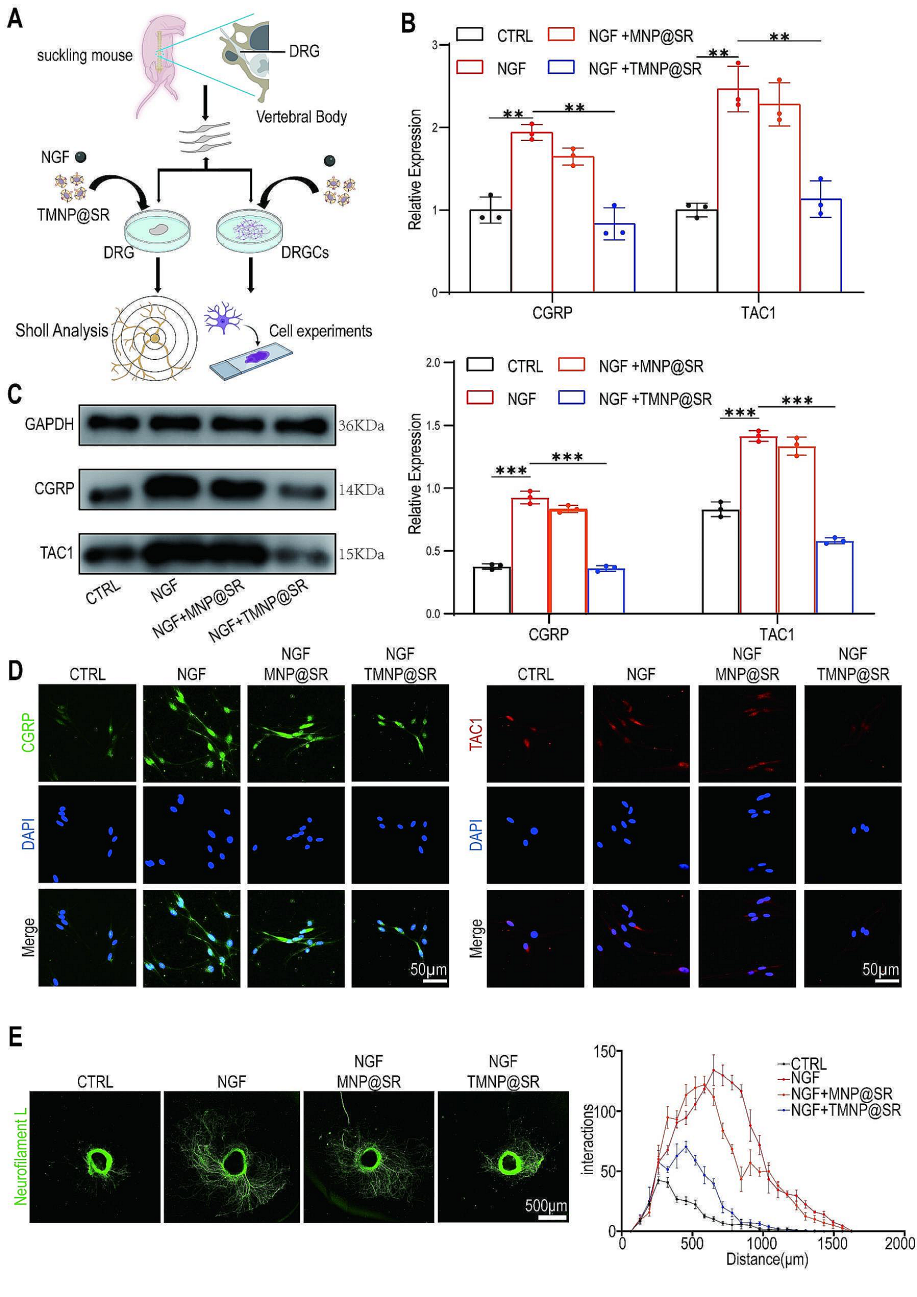
TMNP@SR alleviated DRG axon growth by removing NGF. (**A**) Schematic diagram of the evaluation of TMNP@SR effects on neuron growth and pain sensitivity. (**B**) Relative mRNA expression of the neurogenic mediators (CGRP and TAC1) in DRGCs treated with NGF, NFG + MNP@SR, and NGF + TMNP@SR (biological replicates, Data are presented as the mean ± SD, *n* = 3). (**C**) Representative western blotting plots and densitometric analysis of CGRP and TAC1 in DRGCs (biological replicates, Data are presented as the mean ± SD, *n* = 3). (**D**) Representative immunofluorescence images of CGRP and TAC1 for DRGCs treated with CTRL, NGF, NFG + MNP@SR, and NGF + TMNP@SR. Scale bars: 50 μm. (**E**) Representative immunofluorescence images of neurofilament L in DRGs (biological replicates, Data are presented as the mean ± SD, *n* = 3), and statistical analysis based on Sholl analysis. Scale bars: 500 μm. ns, non-significant, *p < 0.05, **p < 0.01, ***p < 0.001. NGF, nerve growth factor, DRGs, dorsal root ganglions; DRGCs, dorsal root ganglion cells; MNP@SR, TrkA overexpressed macrophage-like SiO_2_-RAPA nanoparticles; TMNP@SR, TrkA overexpressed macrophage-like SiO_2_-RAPA nanoparticles

Discogenic pain is largely attributed to an irritating inflammatory microenvironment [43] in which degenerated intervertebral disc cells can secrete NGF and induce inward growth of neural fibers into intervertebral discs and pain hypersensitivity [10, 44]. Moreover, sensory neurons can produce NGF on their own, forming positive feedback to create an NGF-enriched microenvironment [45, 46]. The removal of NGF by TMNP@SR was previously demonstrated, and the effects of TMNP@SR on neurite growth and irritation were evaluated (Fig. 6A). After treating Dorsal Root Ganglion Cells (DRGCs) with NGF (20 ng/mL) and nanomaterials for one day, the cell supernatant was collected, and the NGF concentration was detected using ELISA. The results showed that the NGF concentrations in the CTLR, NGF, NGF + MNP@SR, and NGF + TMNP@SR groups were 101.3 ± 4.01 pg/mL, 386.9 ± 38.94 pg/mL, 405.8 ± 38.55 pg/mL, and 156.9 ± 6.19 pg/mL, respectively, indicating a reduction of NGF in the microenvironment by TMNP@SR (Figure S5A). Relative mRNA expression levels of two key genes involved in pain sensitivity: calcitonin gene-related peptide (CGRP) and tachykinin 1 (TAC1), were examined using PCR [47]. DRGC pain sensitization increased after treatment with NGF, as evidenced by elevated CGRP and TAC1 expression. The addition of MNP@SR or TMNP@SR alleviated this process, and the effect of TMNP@SR was more evident (Fig. 6B). The protein expression of CGRP and TAC1 was also upregulated by NGF, whereas TMNP@SR reversed these effects (Fig. 6C-D, Figure S5B). DRGs were stained with neurofilament L after treatment with NGF and nanomaterials (MNP@SR and TMNP@SR), and axon growth was estimated using Sholl analysis. Sholl analysis was a quantitative method for the analysis of the growth status of neurons that uses the ImageJ software to quantitatively analyze neuritis [48, 49]. NGF stimulation significantly increased the number of DRGs neurites, whereas the growth of DRGs was inhibited by TMNP@SR (Fig. 6E). These results indicated that TMNP@SR competitively captured NGF to alleviate DRG growth and pain sensitivity.

### TMNP@SR relieved discogenic pain

**Fig. 7:**
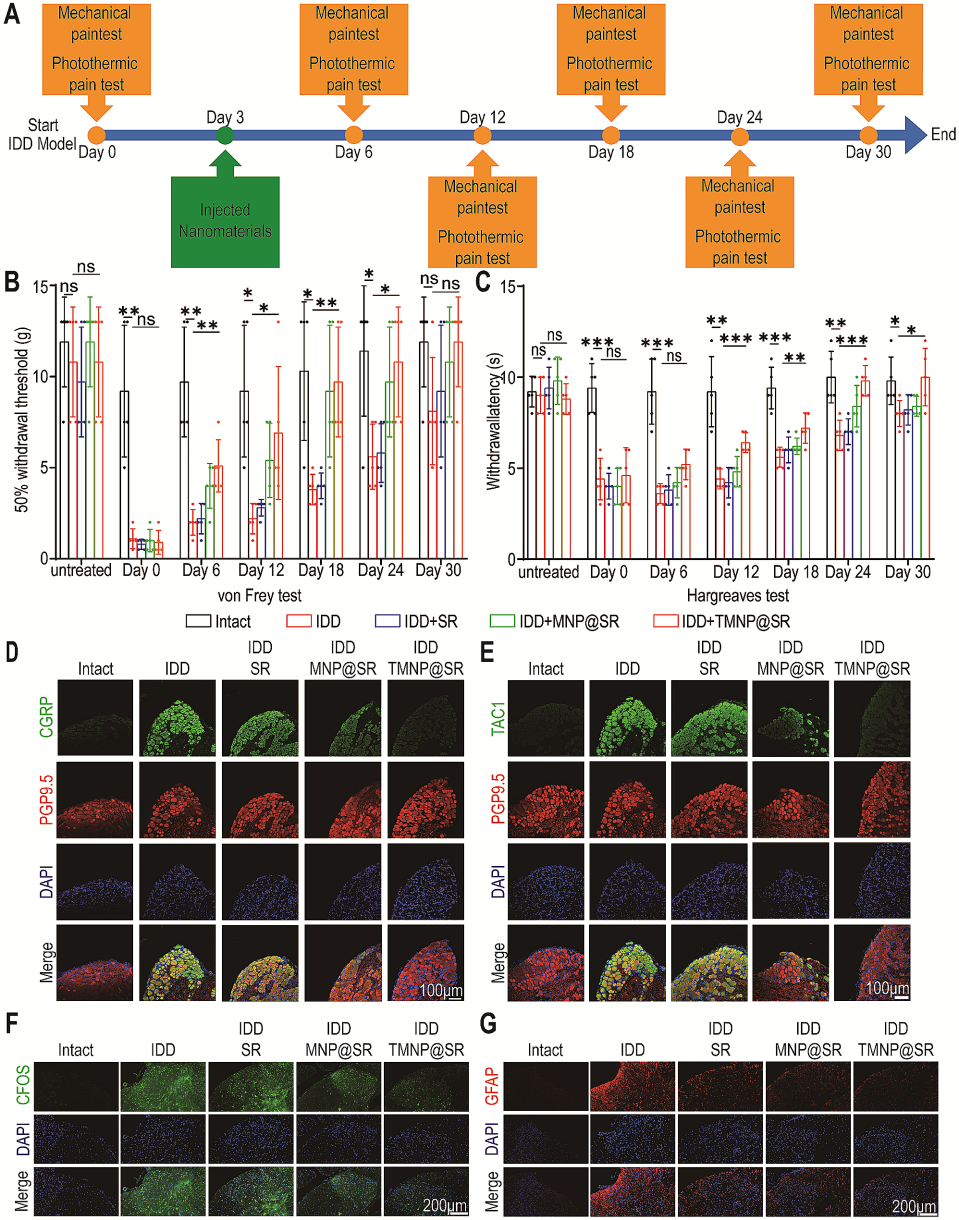
TMNP@SR relieved hyperalgesia in discogenic pain. (**A**) Flow chart of the experiments in vivo. (**B**) The 50% withdrawal thresholds detected by the von Frey test show the mechanical threshold in intact, IDD, IDD + SR, IDD + MNP@SR, and IDD + TMN@SR groups (biological replicates, Data are presented as the mean ± SD, *n* = 5). (**C**) Hargreaves tests detecting the thermal hyperalgesia in response to heat stimulation in different groups (biological replicates, Data are presented as the mean ± SD, *n* = 5). Representative immunofluorescence images of CGRP (**D**) and TAC1 (**E**) of corresponding DRGs (Scale bars: 100 μm). Representative immunofluorescence images of c-FOS (**F**) and GFAP (**G**) of the spinal cords (Scale bars: 200 μm). ns, non-significant, *p < 0.05, **p < 0.01, ***p < 0.001. IDD, intervertebral disc degeneration; SR, mesoporous silica nanoparticles loaded with rapamycin; MNP@SR, macrophage-like SiO_2_-RAPA nanoparticles; TMNP@SR, TrkA overexpressed macrophage-like SiO_2_-RAPA nanoparticles

A rat IDD model was constructed and the nociceptive behavior of rats was measured using a mechanical pain threshold test and Hargreaves test at specific time points. During the observation period, PBS, SR, MNP@SR, and TMNP@SR were injected into the intervertebral discs of rats on day 3 (Fig. 7A). Results showed that compared with the IDD group, the 50% withdrawal threshold in the von Frey test and the withdrawal latency in the Hargreaves test were significantly higher in the IDD + TMNP@SR group at each time point over 30 days (Fig. 7B-C), whereas MNP@SR also showed moderate effects on relieving mechanical and thermal hyperalgesia. After 30 days, the DRGs and spinal cord were collected for immunohistochemical analysis. We located neurons in the DRGs using PGP9.5 and evaluated neuronal pain sensitivity through the fluorescence intensity of CGRP and TAC1 in neurons [50]. CGRP and TAC1 expression in the corresponding DRGs was significantly upregulated in the IDD group compared with the control group and was mostly alleviated in the IDD + TMNP@SR group (Fig. 7D-E, Figure S6A). In addition, the fluorescence intensity of c-Fos (an index of neural activation) and CFAP (a marker of astrocyte activation) in the posterior horn of the spinal cord (sensory neurons)were observed [51, 52]. Compared to the IDD group, c-Fos, and GFAP were downregulated in the posterior horn of the spinal cord in the IDD + TMNP@SR group, indicating suppressed nociceptive transmission in the central nervous system (Fig. 7F-G, Figure S6B). These results indicated that TMNP@SR showed excellent inhibitory effects against hyperalgesia, nerve ingrowth, and activation of nociceptive transmission.

### TMNP@SR relieved IDD by mitigating the pro-inflammatory microenvironment

**Fig. 8:**
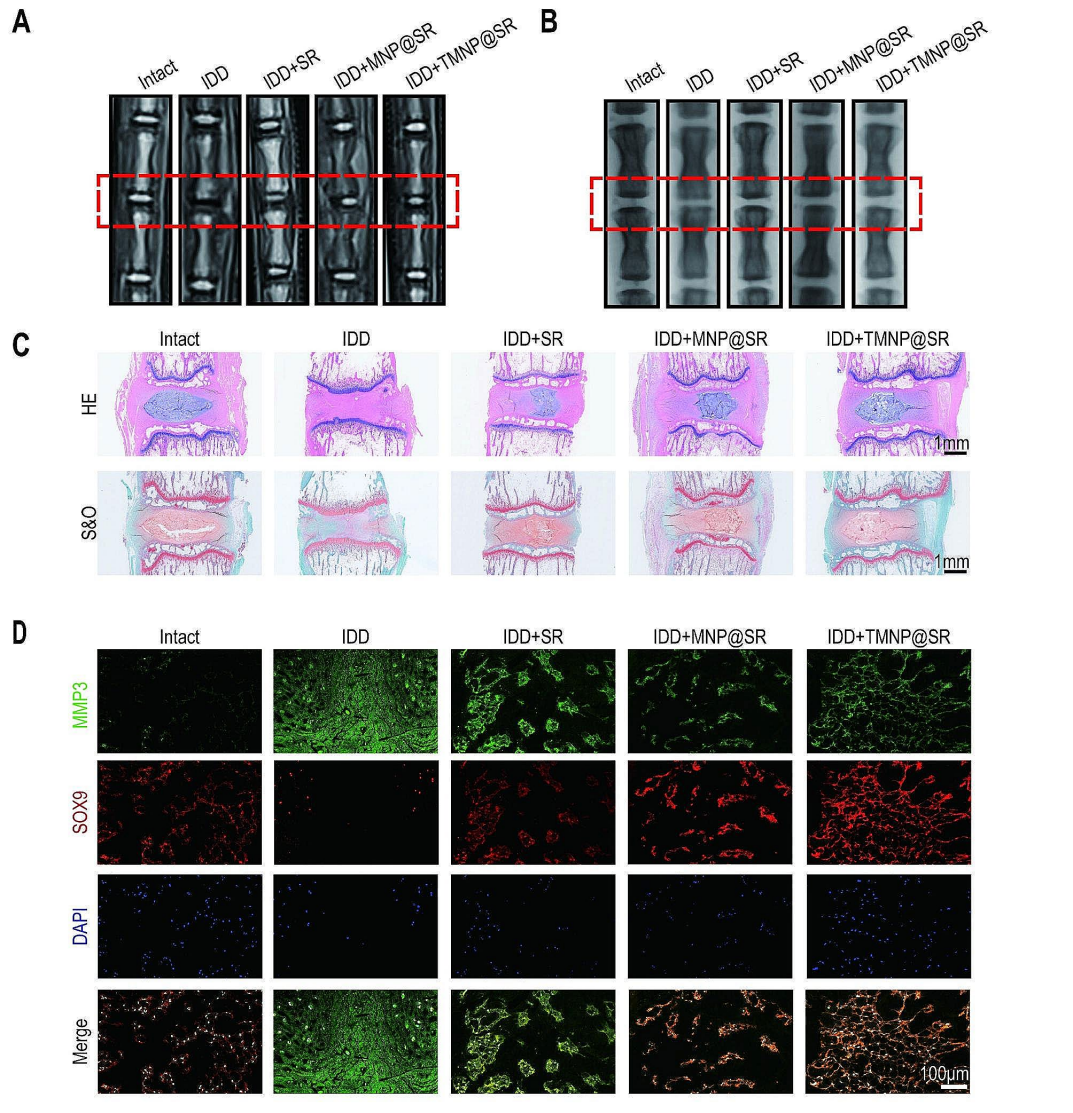
TMNP@SR alleviated intervertebral disc degeneration. (**A**) Representative T2-MRI images for evaluating the relative signal intensity of NP tissues in intact, IDD, IDD + SR, IDD + MNP@SR, and IDD + TMNP@SR groups. (**B**) Representative X-ray images for evaluating the disc height index. (**C**) Representative images of HE and S&O staining of degenerative disc samples in rats treated by SR, MNP@SR, or TMNP@SR. Scale bars: 1 mm. (**D**) Representative immunofluorescence images of rat degenerative disc samples. Scale bars: 100 μm. IDD, intervertebral disc degeneration; SR, mesoporous silica nanoparticles loaded with rapamycin; MNP@SR, macrophage-like SiO_2_-RAPA nanoparticles; TMNP@SR, TrkA overexpressed macrophage-like SiO_2_-RAPA nanoparticles

An IDD model was established via coccygeal vertebral disc puncture and TMNP@SR was injected into the IVDs. MRI T2-weighted imaging was used to evaluate the degree of disc degeneration (Fig. 8A). The modified Pfirrmann grading and relative water content of the intervertebral discs were evaluated based on MRI images (Figure S7A-B). MNP@SR and TMNP@SR reversed the impaired nucleus pulposus hydration in the IDD group Figure S7A). Modified Pfirrmann grading, which indicates the severity of degeneration, indicated that the degenerative grades were significantly lower by MNP@SR and TMNP@SR groups, whereas the therapeutic effect of SR alone was limited (Figure S7B). In addition, TMNP@SR led to most evident relief in puncture-induced disc height reduction (Fig. 8B, Figure S7C). HE and S&O staining showed that TMNP@SR significantly alleviated puncture-induced structural changes, as demonstrated by the restoration of the volume and vacuolated-like matrix in IVDs (Fig. 8C, Figure S7D). The Boos’ scoring of Intact group, IDD group, IDD + SR group, IDD + MNP@SR group and IDD + TMNP@SR group were: 4.4 ± 1.1, 16.0 ± 2.0, 12.2 ± 1.3, 7.4 ± 1.1 and 7.0 ± 1.0. In addition, both MNP@SR and TMNP@SR restored SOX9 expression and decreased MMP3 expression, suggesting an improvement in matrix metabolism (Fig. 8D, Figure S7E).

Next, the inflammatory environment was evaluated and the results showed that after disc puncture, macrophages aggregated and overexpressed iNOS, which was alleviated by treatment with MNP@SR and TMNP@SR (Figure S8A, C). After a puncture, the expression of CD163 in macrophages was slightly upregulated, indicating a tendency to polarize towards M2. After treatment with TMNP@SR, CD163 was highly expressed in the macrophages, resulting in an M1-to-M2 switch (Figure S8B, D). In summary, these results indicate that TMNP@SR effectively alleviates puncture-induced IDD by capturing cytokines and modulating the M1-M2 macrophage balance, thereby maintaining the balance of extracellular matrix metabolism and ultimately alleviating intervertebral disc degeneration.

## Discussion

Nanoparticles are becoming increasingly common in medical research and have significant advantages in terms of efficacy and safety compared with current treatment methods [53, 54]. The design of targeted biomimetic nanomaterials is particularly important for the highly complex biological environment of the body [55]. Cell membrane encapsulation technology was first reported in 2011, in which extracted red blood cell membranes and nanoparticles were co-extruded through a porous membrane, resulting in a membrane-encapsulated nanostructure that could be observed under transmission electron microscopy [56]. Biomimetic nanomaterials, constructed by combining natural cell membranes with core nanoparticles, have the advantage of improving the utilization of nanoparticles in the body and inheriting cell-specific biological functions [31, 57, 58]. When nanoparticles are injected into the body, they can trigger the clearance system, reducing their effectiveness [59]. Homologous cell membranes camouflage nanoparticles, facilitating their retention and effectiveness within the organism [38, 60]. In addition, cell membrane encapsulation technology can be used for cell targeting, such as targeting cancer cells with indocyanine green-loaded cancer cell membrane encapsulated nanoparticles [61], reducing the cellular uptake of macrophage-like cells with platelet membrane encapsulated nanoparticles [31], and developing macrophage membrane-encapsulated nanoparticles for tumor-targeted chemotherapy delivery [62]. Furthermore, membrane encapsulation materials inherit the antigen and receptor functions of the source cells and play a role in delivering tumor antigens and promoting anticancer immunity with membrane-encapsulated nanoparticles [63], using mannose-modified cancer cell membrane-encapsulated adjuvant nanoparticles for cancer vaccine administration [64], T cell membrane-encapsulated nanoparticles inheriting CD4 receptors to neutralize HIV infectivity [65]. Therefore, the cell membrane encapsulation technology provides a new treatment strategy for intervertebral disc inflammation and pain through cell mimicry and targeting [59]. In this study, a nanodecoy, TMNP@SR, with an engineered macrophage membrane as the shell and rapamycin-loaded mesoporous silica as the core was established. Excellent cell-specific targeting effects of TMNP@SR towards macrophages rather than other intervertebral disc cells were observed.

Damage to the peripheral fibrous ring or endplate leads to infiltration and polarization of macrophages, which disrupts the immune balance [7]. Proliferating clusters of macrophages respond to cytokines in the extracellular microenvironment of IDD cells, resulting in significant phenotypic and functional changes [66]. Macrophages polarize into M1 or M2 cells based on their response to stimuli in the microenvironment. M1 macrophages are present in inflammatory environments primarily driven by Toll-like receptors (TLR) and interferon signaling and produce high levels of inflammatory cytokines such as TNF-α, IL-1β, IL-6, IL-12, and IL-23, further exacerbating the progression of IDD [67]. Anti-inflammatory effects of M2-polarized macrophages have been reported in various diseases. For instance, human umbilical cord mesenchymal stem cell-derived extracellular vesicles induce M2 polarization in the treatment of osteoarthritis [68]. Specific knockout of Xbp1 in macrophages exhibits M2 polarization in regulating hepatic steatosis [69], and engineered exosomes induce M2 polarization in macrophages for the treatment of rheumatoid arthritis [70]. Therefore, restoring the balance between M1 and M2 macrophage polarization is a novel approach to halting the progression of IDD.

RAPA is a naturally occurring antibiotic originally discovered in bacteria [71]. RAPA is a specific inhibitor of mTOR, a serine/threonine kinase that is a major regulator of cell growth and metabolism [72, 73]. Autophagy is a biological process that assists cells in self-cleaning by removing damaged macromolecules and organelles to maintain cellular health and function. mTOR typically inhibits the autophagic process [73]. RAPA, an inhibitor of mTOR, activates the autophagic process in cells [74]. RAPA has gained attention as a therapeutic drug for IDD. Recent research suggests that mTOR is a new therapeutic target for restoring immune dysregulation and alleviating inflammatory processes [75]. RAPA has been demonstrated to induce cell activation of the autophagic process and exhibit strong anti-inflammatory effects by promoting autophagy [76]. RAPA-induced autophagy has shown promise in alleviating various health conditions [77]. Biomimetic drug delivery systems that administer RAPA locally are effective in reducing vascular inflammation [78]. Thus, RAPA may ameliorate age-related pathological changes by inhibiting age-related inflammation [79].

Local treatment with RAPA can also help avoid the systemic administration of RAPA and mitigate many adverse effects, such as high triglyceride levels, high cholesterol levels, and interstitial lung disease [80, 81]. Recent studies have suggested that sustained release of RAPA effectively promotes M2 polarization of macrophages [82]. Furthermore, tumor-associated macrophages promote M2 polarization and accelerate tumor development via autophagic waves [83]. In this study, RAPA alone promoted macrophage transformation into the M2 phenotype, but the efficiency was low. However, encapsulating SR within TMNP improved the efficiency of macrophage transformation into the M2 phenotype. In vivo experiments also confirmed that compared to the SR group, the TMNP@SR group showed a significant increase in the proportion of CD68+ and CD163+ double-positive cells. TMNP@SR also demonstrated a higher efficiency in autophagy activation. These results suggest that TMNP@SR effectively promoted autophagy activation and M2 macrophage transformation by delivering nanocores to macrophages. Additionally, RAPA also has the ability to reduce macrophage proliferation [78] and enhance their phagocytic functions [84, 85]. However, the cell compatibility experiments in this study have demonstrated that TMNP@SR, loaded with a RAPA concentration of 80 µg/ml, did not lead to significant macrophage cell death. Instead, it effectively triggered autophagy and M2 polarization, highlighting the excellent biological functionality of the developed material at a safe dosage.

Intervertebral discogenic pain is closely associated with the inflammatory microenvironment of IDD [86]. In an inflammatory environment, intervertebral disc and immune cells produce NGF, which irritates pain-related ion channels in DRG. Depolarization of these channels may contribute to discogenic and radicular pain [10]. Neurotrophic factor receptors include two main types: the tyrosine kinase receptors Trks and the tumor necrosis factor receptor superfamily member p75^NTR^. Among these, TrkA1 has high affinity for NGF and plays a significant role in its biological binding. The neurotrophic factor antagonist, tanezumab, has been used to treat lower back pain. However, it can lead to adverse effects, such as joint pain, sensory abnormalities, decreased sensation, and osteonecrosis [87]. Therefore, the development of biomimetic NGF antagonists to enhance treatment efficacy and reduce complications is imperative. By overexpressing TrkA1 on macrophage membranes, TMNP was obtained, which effectively sequestered NGF in the microenvironment and competitively reduced NGF binding to neurons, thereby inhibiting axonal growth and the release of pain factors.

Although there are significant differences in the original measurement values between intervertebral discs of different species, such as the intervertebral discs of mice and rats being much smaller than those of humans, while the intervertebral discs of cows and pigs are relatively larger. However, through the standardization of geometric parameters, similarities in mechanical properties can be found. This provides a theoretical basis for using animal models to study human intervertebral disc disease and helps explain experimental results and select appropriate models [88]. The main limitation for this study is that in-vivo experiments were currently limited to small animals—rats. Validation with larger animals, such as monkeys, dogs, or sheep is required for further translational research.

## Conclusion

In conclusion, we have constructed biomimetic nanomaterials with a SiO_2_-RAPA (SR) core, which is enveloped by cell membranes sourced from macrophages that overexpress TrkA1 (represented as TMNP@SR, as shown in Fig. 1A). TMNP@SR exhibits an antigenic exterior nearly identical to that of macrophages. Therefore, TMNP@SR not only possesses the capability of being specifically taken up and engulfed by macrophages but also inherits the ability to adsorb LPS and inflammatory factors like macrophages. Simultaneously, SR, by means of the sustained release of RAPA, plays a role in modulating macrophage-mediated immune microenvironments. The overexpression of TrkA1 on macrophages increases the number of NGF receptors on their membranes, imparting TMNP@SR with competitive binding capacity for NGF. These multiple functions collectively achieve effective intervention in the IDD inflammatory environment, offering a potential therapeutic strategy for LBP.

## Materials and methods

### Transfection

A Lentivirus-mediated overexpression vector of TrkA1 was generated using GeneChem (CHN). The Lentivirus was ultracentrifuged, concentrated, validated, and added to the complete medium. After infection, the cells were selected with puromycin (2 µg/mL) (Beyotime, CHN) for 9 days. Live cells were then validated and continuously cultivated.

### Cell membrane extraction

The murine J774A.1 cell line (Haixing Bioscience, China) was selected as the macrophage line. Macrophages were maintained in Dulbecco’s Modified Eagle Medium (DMEM) (Gibco, USA) supplemented with 10% fetal bovine serum (FBS) (Gibco) and 1% penicillin–streptomycin (P/S) (Gibco). The methods were based on those described in the literature for cell membrane extraction [89]. Cell scrapers (Thermo Fisher Scientific, USA) were used to scrape off the macrophages and obtain cell suspensions, and an ultrasound machine (Sonics, Denmark) was used for 30 s to destroy the cells. The cell suspension was then centrifuged at 3000 × g for 5 min. The supernatant was collected and centrifuged at 20,000 × g for 30 min. Finally, the supernatant was centrifuged at 100,000 × g for 40 min, and the cell membrane precipitates were collected for subsequent experiments.

### Preparation and characterization of nanoparticles

Ten milligrams of RAPA (MedChemExpress, USA) in DMSO were added to a PBS solution containing mesoporous SiO_2_ (XFNANO, CHN) to obtain the SR nanoparticles. The morphologies of the SiO_2_ and SR were confirmed using SEM (HITACHI, JPN). The nanoparticles were coated with macrophage membranes using an extruder (HandExtruder, 1mL; Genizer LLC, USA). The prepared nanoparticles were then freeze-dried. The process remained consistent for macrophage membranes that overexpress the TrkA1 gene and empty vector-transfected macrophage membranes. Then, 2% phosphotungstic acid was used to negatively stain nanoparticles and observe their morphology using transmission electron microscopy (TEM) (HITACHI). A dynamic light scattering detector (Zetasizer Nano ZS90, Malvern Panalytical, UK) was used to measure the hydrodynamic size and zeta potential of nanoparticles.

### Drug loading and drug release

To assess the drug loading capacity of nanoparticles, RAPA solutions ranging from 0 to 100 µg/mL were initially prepared, and subsequently, a calibration curve was constructed. The quantification of RAPA content in excess solvent prior to drug loading was performed by measuring the absorbance at its maximum absorption wavelength (λ_Max) of 280 nm using UV-visible spectroscopy [90–92]. The formula for calculating drug loading is as follows: DL (%) = Mr/Ms × 100% (where DL is the drug loading capacity of SiO_2_; Mr is the mass of RAPA encapsulated in SiO_2_, and Ms is the total mass of SiO_2_). For drug release experiments, the nanoparticle solution was dispersed in phosphate-buffered saline (PBS) at different pH values and incubated at 37 °C; equal volumes of mixed solutions were collected at different time points and replaced with equal volumes of release media. The RAPA contents of the mixed solutions collected at different time points were detected using UV-visible light. In subsequent experiments, the concentration of the nanomaterials indicated the RAPA concentration.

### Encapsulation ratio

The encapsulation ratio was determined by fluorescence dot matrix experiments [93]. Next, we measured the encapsulation ratios of the nanomaterials. The membrane was labeled with DiO (green). SiO_2_ and SR were labeled with CY3 (red, Yeasen). The encapsulation ratio was determined by calculating the ratio of the number of particles showing both green and red fluorescence to the total number of red fluorescent particles.

### Extraction and culture of intervertebral disc cells

The intervertebral discs were obtained from the coccygeal vertebras of 4 to 6 week-old rats. The nucleus pulposus, annulus fibrosus, and cartilage endplate of the rat intervertebral disc were separated. To extract NPCs, the nucleus was cut with scissors and digested with 0.25% type II collagenase (Yeasen, China) for 30 min and centrifuged. The cell precipitates were cultured with DMEM Nutrient Mixture F-12 (DMEM/F-12) (Gibco) supplemented with 10% FBS and 1% P/S. To extract AFCs, the annulus fibrosus was cut with scissors, digested with 0.25% type II collagenase for 4 h, then digested with 0.25% trypsin-EDTA (Gibco) for 15 min, and centrifuged. The cell precipitates were cultured with DMEM/F-12 supplemented with 10% FBS and 1% P/S. To extract CECs, the cartilage endplate was cut with scissors, digested with 0.25% type II collagenase overnight, and then digested with 0.25% trypsin-EDTA for 15 min. After centrifugation, the cell precipitates were cultured with DMEM/F-12 supplemented with 10% FBS and 1% P/S.

### Extraction and culture of DRGCs

DRGs were obtained from the spines of 2 to 6 days-old rats. DRG was digested with 0.25% trypsin-EDTA for 15 min and with 0.2% type II collagenase for 20 min. After termination of digestion, the cell precipitates were cultured with DMEM/F-12 supplemented with 10% FBS and 1% P/S for 1 day. The cells were then cultured with rat dorsal root neuron cells in a complete culture medium (Procell, CHN) containing cytarabine (5 µΜ/mL) (MedChemExpress) for 2 days for subsequent experiments.

### Cell counting Kit‑8 analysis

The cells were plated in a 96 well plate (5 × 10^3^ cells per well). After the cells were attached completely, they were co-cultured with the kit material for 1 day. A suitable amount of reagent from the Cell Counting Kit-8 (Dojindo, Kumamoto, JAP) (10 µL) was added to the cells and the cells were further cultured for 4 h. Optical density (OD450) values were analyzed using a Multimode Plate Reader (PerkinElmer, USA). Cell viability was calculated using the optical density, with the control group normalized to 1.

### Live/dead staining assay

Macrophages were seeded in 96-well culture plates (1.5 × 10^5^ cells per well). After the cells were attached completely, they were co cultured with the kit material for 1 day. Live/dead staining was performed using the Calcein/PI assay kit (Beyotime). Briefly, cells were incubated with calcein and propidium iodide (PI) for 12 min. After washing with PBS, cells were imaged under a microscope (Olympus, Tokyo, JAP).

### Hemolytic nanoparticle test

Whole blood was collected from the rats, centrifuged at 1200 × g for 10 min, and the lower layer of the precipitate (red blood cells) was collected. The red blood cells were co-cultured with different concentrations of nanomaterials at 37 °C for 5 h. Red blood cells were co cultured with pure water as a positive group. After 5 h, the mixture was centrifuged at 12,000 rpm for 10 min and the color of the supernatant was observed. The supernatant was collected, and its absorbance was measured at 540 nm.

### Enzyme-linked immunosorbent assay (ELISA)

Nanomaterials were co-cultured with LPS (MedChemExpress, HY-D1056), NGF (Peprotech, USA), TNF-α (Peprotech), IFN- γ (Peprotech), IL-1β (Peprotech), and IL-6 (Peprotech, 400-06) for 1 h. The mixture was centrifuged at 100,000 × g for 40 min and the supernatant was collected. Residual LPS (Meimian, CHN), NGF (Meimian), TNF-α (Neobioscience, CHN), IFN- γ (Neobioscience), IL-1β (Neobioscience) and IL-6 (Neobioscience) was detected using ELISA kits according to manufacturers’ instructions.

### Flow cytometry

For flow cytometry detection of nanomaterial-targeting DiD (Yeasen) was used to stain NPCs, DiI (Yeasen) to stain AFCs, Hoechst 33,342 (Yeasen) and DiI were used to stain macrophages, and Hoechst 33,342 and DiD were used to stain CECs. TMNP@SR loaded DiO (Yeasen). After treating several cells with TMNP@SR for 6 h, the cells were analyzed using flow cytometry. To detect macrophage polarization by flow cytometry, macrophages were placed in flow cytometry tubes and washed three times with PBS. To detect specific markers, macrophages were incubated with anti-CD206 (Biolegend, USA, 141,737) and anti-CD86 (Biolegend,105,019) in the dark at 4 °C for 30 min. The cells were then analyzed using flow cytometry (BD Biosciences, USA). For flow cytometry detection of apoptosis, NPCs were placed in a flow cytometry tube and washed three times with PBS. NPCs were treated with the Annexin V-PI cell apoptosis detection kit (Vazyme, CHN) in darkness at 4 °C for 30 min. The cells were analyzed by flow cytometry.

### Quantitative real-time polymerase chain reaction (qRT-PCR)

Total RNA from the cultured cells was purified using an RNA-easy Isolation Reagent (Vazyme, CHN). Subsequently, a first-strand cDNA synthesis kit (Yeasen) was used to produce cDNA from total RNA. Glyceraldehyde 3-phosphate dehydrogenase (GAPDH) was used as an internal control. The primer sequences are listed in Table 1. Target gene transcripts were detected using SYBR Green quantitative real-time polymerase chain reaction (PCR) Super Mix Plus (Yeasen) on a real-time PCR system (Bio-Rad, USA). The procedure was set as 45 cycles of 95 °C for 20 s, 60 °C for 30 s, followed by 75 °C for 30 s. Finally, the 2^−ΔΔ^CT method was used to measure the relative expression of target genes.

**Table 1:** The PCR primers utilized in this study

Gene	Sense (5′–3′)	Antisense (5′–3′)
GAPDH-M	ATTCAACGGCACAGTCAA	TTAGTGGGGTCTCGCTCC
TrkA1-M	TCAACAACGGGAACTACA	GAGAAGGAGACAGGGATG
ARG1-M	CAGTCTGGCAGTTGGAAGC	TGGTTGTCAGGGGAGTGTT
CD206-M	ACCCAAGGGCTCTTCTAA	TGGCCTCTTGAGGTATGT
IL-10-M	TTCAAACAAAGGACCAGC	GGATCATTTCCGATAAGG
INOS-M	TGGAGCGAGTTGTGGATTG	TGAGGGCTTGGCTGAGTGA
TNF-α-M	GCGGTGCCTATGTCTCAG	TCCTCCACTTGGTGGTTT
IL-6-M	ATTTCCTCTGGTCTTCTGG	TGGCTTTGTCTTTCTTGTTA
GAPDH-R	GCAAGTTCAACGGCACAG	CTCGCTCCTGGAAGATGG
CGRP-R	CCTGGTTGTCAGCATCTT	CTCAGCCTCCTGTTCCTC
TAC1-R	TGACCAAATCAAGGAGGC	CAAAGAACTGCTGAGGCT

M indicates mouse; R indicates rat

### Western blot assay (WB) and coomassie brilliant blue

RIPA lysis buffer (HYCEZMBIO, CHN) was used to purify proteins from the cells. The ExKine™ Membrane and Cytoplasmic Protein Extraction kit (Abbkine, USA) was used to purify cell membrane and nanoparticle proteins. Protein concentrations were determined using a BCA protein assay kit (Beyotime). SDS-PAGE sample loading buffer (Yeasen) was added to the proteins and heated in a boiling water bath for 10 min for full denaturation. Used Easy PAGE^®^ Gel Fast Preparation Kit (Seven, JAP) to create a gel and protein samples were electrophoresed. Western blotting: Using a transfer membrane (Millipore, USA), transmembrane electrophoresis was performed at a flow rate of 200 mA. The transfer membranes were incubated with primary antibodies, anti-GAPDH (Proteintech, USA, 10494-1-AP), anti-Na^+^K^+^-ATPase (ABclonal, CHN, AP1065), anti-Flag (abcam, UK, ab1162), anti-CD14 (ABclonal, A5737), anti-TLR4 (ABclonal, A5258), anti-F4/80 (ABclonal, A23788), anti-CD120a (ABclonal, A1540), anti-LC3B (Cell Signaling Technology, USA, #12,741), anti-beclin1 (ABclonal, A21191), anti-MMP3 (ABclonal, A1202), anti-MMP13 (ABclonal, A11148), anti-SOX9 (ABclonal, A19710), anti-Col2A1 (ABclonal, A21059), anti-CGRP (ABclonal, A5542), or anti-TAC1 (Proteintech, 13839-1-AP) at 4 °C overnight. A secondary antibody, anti-rabbit IgG (ABclonal, AS014), was incubated at room temperature for 1 h. The expression of each protein was assessed using a chemiluminescence instrument (Bio-Rad). Coomassie brilliant blue: The electrophoretic gel was stained with Coomassie blue staining solution for 2 h and rinsed with a decolorization solution for 4 h. The gel was imaged using a chemiluminescence instrument.

### Cellular immunofluorescence

The cells were inoculated onto a slide and treated according to the experimental protocol. Cells were treated with 4% paraformaldehyde (Biosharp, CHN) for 15 min. After fixation, the cells were incubated in a PBS solution containing 0.5% Triton (Biosharp) for 15 min for permeation. The cells were then sealed with goat serum (Boster, CHN) for 1 h. The cells were incubated with primary antibodies, anti-Flag, anti-MMP3, anti-MMP13, anti-SOX9, anti-Col2A1, anti-Neurofilament L (ABclonal, A20269), anti-CGRP, or anti-TAC1 at 4 °C overnight. Secondary antibodies, 594-conjugated goat anti-rabbit IgG (ABclonal, AS039) or 488-conjugated goat anti-rabbit IgG (ABclonal, AS053), were incubated at room temperature for 1 h. The cells were incubated with DAPI (Yeasen) at room temperature for 15 min. After incubation, mounting medium (Solarbio, CHN) was added to the cell slide, and the cells were imaged using a microscope.

### Autophagy double-labeled Adenovirus (mRFP-GFP-LC3)

Autophagic flux was evaluated using mRFP-GFP-LC3 (Hanbio, CHN). The co-localization of GFP and RFP fluorescence indicated autophagosomes, and only RFP fluorescence indicated autolysosomes. Macrophages were transfected with mRFP-GFP-LC3 for 12 h and then treated with RAPA, SR, or TMNP@SR for 1 day. Macrophages were incubated with 4% paraformaldehyde for 15 min. The cells were then incubated with DAPI at room temperature for 15 min. After incubation, a mounting medium was added to the macrophages. Finally, images were captured using a confocal microscope (Olympus, Tokyo, Japan), and the number of puncta per cell was calculated.

### Animal experiments

All animal experiments conducted in this study were approved by the Ethics Committee of the Tongji Medical College, Huazhong University of Science and Technology ([2023] IACUC Number 3465). We used male Sprague-Dawley (SD) rat, aged 7 weeks, with a weight of around 210 g. The IDD model was established via coccygeal vertebral disc puncture, and the coccygeal vertebrae Co5/6 were located by manual palpation and counting and confirmed by a trial radiograph [94]. We used a 20-gauge needle, inserted it into the intervertebral disc at a depth of 6 mm, rotate 360°, held for 30 s, and then remove it. SD rats were divided into five groups: intact, IDD, IDD + SR, IDD + MNP@SR, and IDD + TMNP@SR. In animal experiments, the number of SD rats in each group is 5. After establishing the animal model, the nanomaterials were injected on the third day. The dose for each injection of nanomaterials was 5 µL at the concentration of 80 µg/mL by the Microlite Syringe Model 65 RN with a 29-gauge needle. There was no significant difference in the weight of experimental animals between groups. After 30 days, the DRG and spinal cord of the rats were removed and fixed with 4% paraformaldehyde. The coccygeal vertebra of rats were removed, fixed with 4% paraformaldehyde, and decalcified for 1 month. Processed specimens were embedded in paraffin and sliced for subsequent experiments. The intervertebral disc was sliced along the coronal plane through the midpoint of the horizontal plane of the intervertebral disc. The thickness of the tissue slice was 4 μm.

### Mechanical pain threshold test and Hargreaves test

Before the start of the test, make the rats accustomed to the test environment for at least 20 min, which will minimize motor activity and stress induced analgesia during the test period. Five researchers who were not aware of the specific grouping in these experiments evaluated rats’ painful behavior using the Mechanical Pain Threshold Test and Hargreaves Test. Mechanical pain threshold and Hargreaves tests were performed before surgery and on days 0, 6, 12, 18, 24, and 30 postoperatively. Mechanical pain threshold test [95]: The rats were individually housed in a six-section enclosure designed with a metal mesh floor and a lid featuring air vents, allowing for a 20 min acclimation period to reduce exploratory behavior. The experiment commenced with the application of a 2-gram filament to the base of the tail on the ventral side, exerting enough pressure to slightly bend the filament for a maximum duration of 6 s. A positive response was recorded if the rat immediately or within the 6-second window exhibited behaviors such as tail retraction, licking, or shaking in response to the filament. Conversely, a lack of such a response was noted as a negative outcome. This procedure was repeated five times using the 2-gram filament. If the filaments cause a reaction in rats, they were treated with lighter filaments, and the experiment stops when five consecutive attempts fail to elicit a reaction in the rats. If no response was observed with the 2-gram filament, the testing proceeded with filaments of increasing weight until all five attempts resulted in a response. A minimum interval of 2 min was maintained between each test to ensure accuracy. The mechanical pain threshold was determined using the 50% value of the filament weight at which a response was observed. Hargreaves test [95]: The rats were placed within an acrylic chamber to acclimate for a duration of 20 min. Heat was then applied using an infrared light source, and the latency of the rats’ responses was meticulously recorded. These responses included tail retraction, licking, biting, and tail shaking. The testing head of the analgesiometer was positioned beneath the tail, with the beam precisely aimed at the ventral surface of the tail, opposite the area of injury. The intensity of the light source was set to 50% of its maximum output. Upon the rat’s response, the light source was immediately reduced to ‘idle’ intensity, and the heat source was deactivated to prevent tissue damage, with a 20-second cut-off period in place as a precaution. Each rat underwent four trials on each experimental day, with a minimum interval of 5 min between trials to ensure the animal’s well-being and to avoid sensitization. The withdrawal latency for each rat was determined by averaging the four recorded latencies.

### Magnetic resonance imaging (MRI) and radiography

MRI: A 3.0 T MRI scanner (GE Medical Systems, UK) was used to obtain T2-weighted mid-sagittal sections of the coccygeal vertebra. Five spinal surgeons who were blinded to this study evaluated the modified Pfirrmann grading and relative water content of the intervertebral disc [96]. Radiography: Radiographs were used on the x-ray to obtain midsagittal sections of the coccygeal vertebra. Five spinal surgeons who were blinded to this study evaluated the disc height index (DHI) of the intervertebral disc [97]. 

### Histological evaluation

Slices of rat specimens were stained with hematoxylin and eosin (HE) (Solarbio) and Safranin O-fast green (S&O) (Solarbio). Five independent researchers evaluated the histological grading of rat slices by Boos scoring system [94, 98].

### Tissue immunofluorescence

The rat slices were incubated in PBS solution containing 0.5% Triton for 30 min for permeation. The cells were then incubated with goat serum for 1 h. The slices were incubated with primary antibodies: anti-CGRP, anti-TAC1, anti-PGP9.5 (Santa cruz, USA, sc-271,639), anti-CFOS (ABclonal, A24619), anti-GFAP (ABclonal, A0237), anti-MMP3, anti-SOX9 (Santa Cruz, sc-166,505), anti-iNOS (Abcam, ab178945), anti-CD163 (Abcam, ab182422), and anti-CD68 (Santa cruz, sc-17,832) at 4 °C overnight. Secondary antibodies, 594-conjugated goat anti-rabbit IgG, 488-conjugated goat anti-rabbit IgG, or 594-conjugated goat anti-mouse IgG (ABclonal, AS054), were used at room temperature for 2 h. Then, the slices were incubated with DAPI at room temperature for 15 min.

### Statistical analysis

All experiments were repeated on at least three biological replicates, and data are expressed as means ± standard deviation (SD). The datasets were compared statistically by analyzing their normality and variance. The statistical significance between the two groups was calculated using Student’s t-test (two-tailed). One-way analysis of variance (ANOVA) was employed to compare differences among multiple groups, and Tukey’s post hoc test was utilized for multiple post hoc comparisons to ascertain the significance of differences between the groups after the one-way ANOVA. In cases where the data did not conform to a normal distribution, the Kruskal-Wallis H-test or the Mann-Whitney U-test was applied. Statistical analyses were performed using the SPSS software package (IBM SPSS software package 18.0), and statistical charts were drawn using GraphPad Prism 7 software (GraphPad Software Inc., San Diego, CA). *p* < 0.05 was considered statistically significant.

### Electronic supplementary material

Below is the link to the electronic supplementary material.

Supplementary Material 1

## Data Availability

No datasets were generated or analysed during the current study.
