# Supplementary material for: A structured biomimetic nanoparticle as inflammatory factor sponge and autophagy-regulatory agent against intervertebral disc degeneration and discogenic pain
Source: J Nanobiotechnology. 2024 Aug 14;22:486. doi: 10.1186/s12951-024-02715-x (PMC11323362; doi:10.1186/s12951-024-02715-x)
Supplement: Supplementary file 1 — Supplementary Material 1 [file 12951_2024_2715_MOESM1_ESM.docx]

**Figure legends for Supplementary materials**


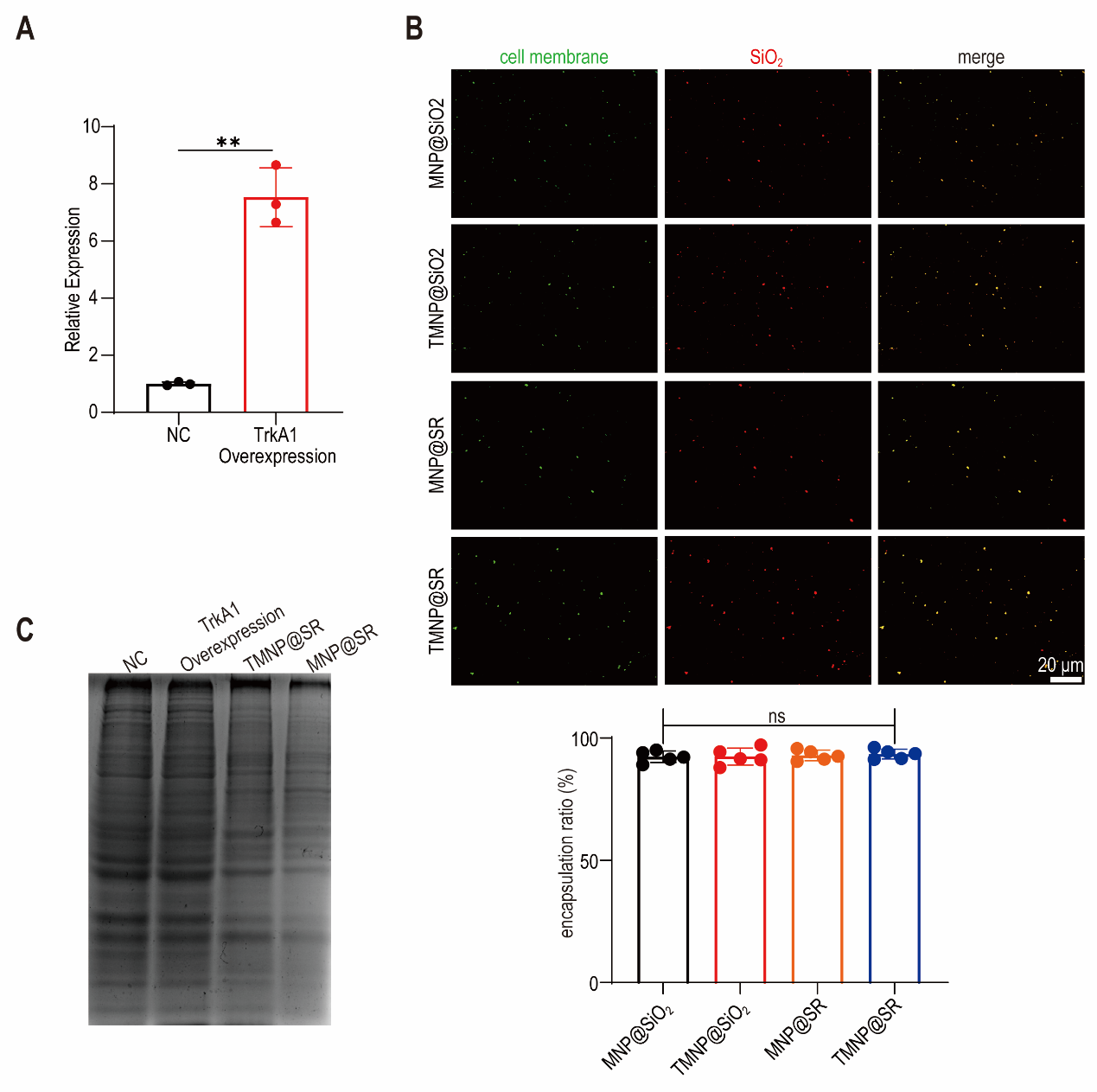


**Figure S1.** The TrkA1 expression in macrophages and nanomaterials. (A) Relative mRNA expression of TrkA1 between NC and TrkA1 Overexpression by quantitative RT-PCR analysis (biological replicates, Data are presented as the mean ± SD, n = 3). (B) Membrane was labeled with DiO (green). SiO_2_ and SR was labeled with CY3 (red). The encapsulation ratio was directly determined by calculating the ratio of the number of particles showing green and red fluorescence to the number of red fluorescence particles (biological replicates, Data are presented as the mean ± SD, n = 5). Bar: 20 μm. (C) Representative Coomassie Blue staining plot indicated the membrane protein spectrum for the macrophages transfected with control vectors (NC), macrophages overexpressing TrkA1, MNP@SR and TMNP@SR. ns, non-significant, *p < 0.05, **p < 0.01, ***p < 0.001. NC, negative control. MNP@SR, macrophage-like SiO_2_-RAPA nanoparticles; TMNP@SR, TrkA overexpressed macrophage-like SiO_2_-RAPA nanoparticles; MNP@SiO_2_, macrophage-like SiO_2_ nanoparticles; TMNP@SiO_2_, TrkA overexpressed macrophage-like SiO_2_ nanoparticles.


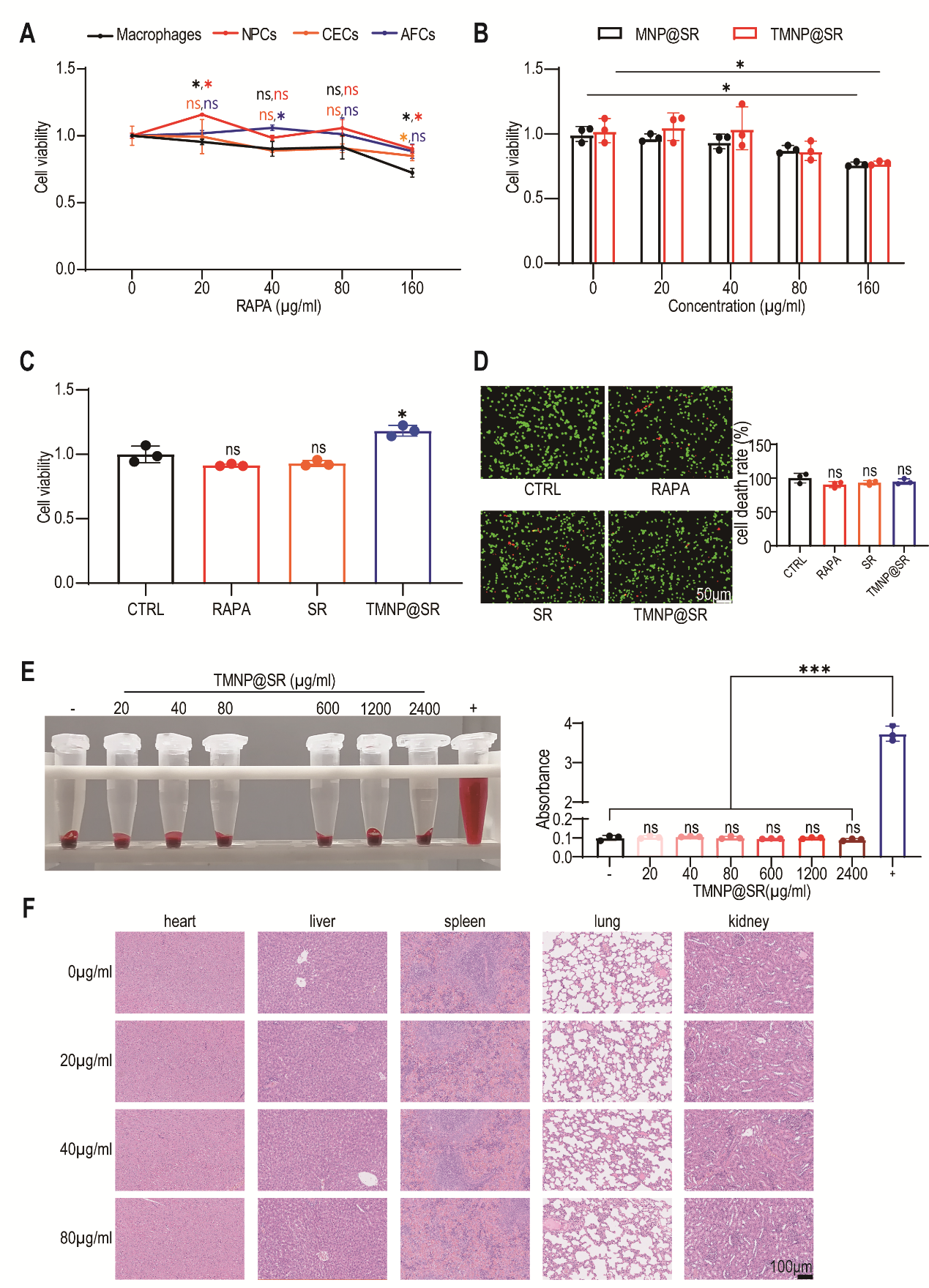


**Figure S2.** Biocompatibility of TMNP@SR. (A)The viability of NPCs, CECs, AFCs, and macrophages treated with RAPA was measured by CCK-8 analysis (biological replicates, Data are presented as the mean ± SD, n = 3). (B) The viability of nucleus pulposus cells treated with MNP@SR and TMNP@SR (biological replicates, Data are presented as the mean ± SD, n = 3). (C) The viability of macrophages treated with CTRL, RAPA, SR, and TMNP@SR (biological replicates, Data are presented as the mean ± SD, n = 3). (D) The impact of CTRL, RAPA, SR, and TMNP@SR on the viability of macrophages detected by the cell live/ death experiment; green represents live cells while red represents dead cells. Scale bar: 50 μm. (E) s assay of different concentrations of TMNP@SR: The absorbance value of the supernatant at 540 nm after 8-hour incubation was quantitatively analyzed (biological replicates, Data are presented as the mean ± SD, n = 3). (F) Representative hematoxylin and eosin (HE) staining images of heart, liver, spleen, lung, and kidney after intravenous injection with different concentrations of TMNP@SR. Bar: 100 μm. ns, non-significant, *p < 0.05, **p < 0.01, ***p < 0.001. SR, mesoporous silica nanoparticles loaded with rapamycin; MNP, macrophage-like nanoparticles; MNP@SR, macrophage-like SiO_2_-RAPA nanoparticles; TMNP@SR, TrkA overexpressed macrophage-like SiO_2_-RAPA nanoparticles; NPCs, nucleus pulposus cells; AFCs, annulus fibrosus cells; CECs, endplate cells.


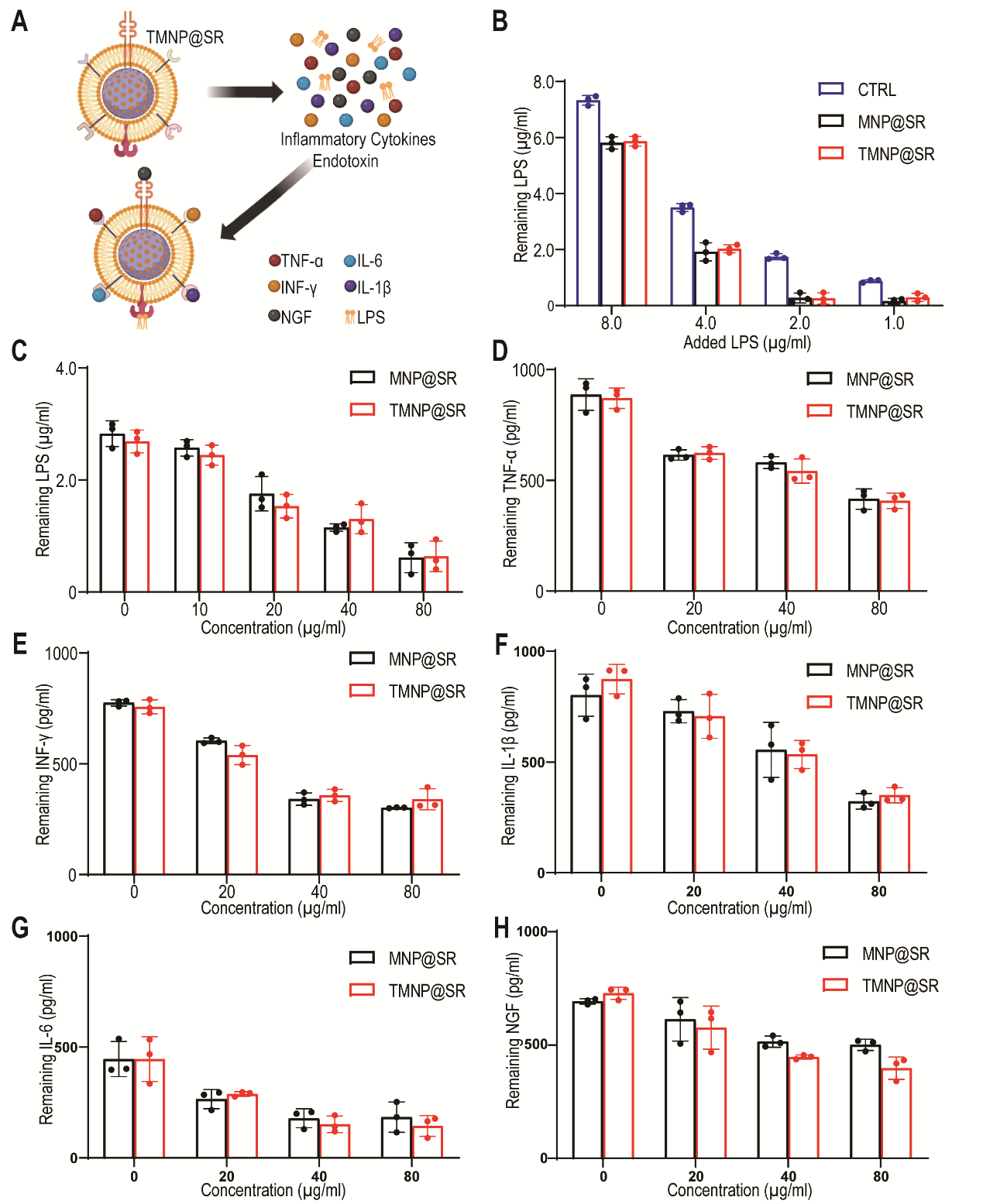


**Figure S3.** TMNP@SR as a decoy for NGF, LPS, and proinflammatory cytokines. (A) Schematic representation of TMNP@SR removing NGF, LPS, and proinflammatory cytokines. (B) The remaining concentration of NGF after incubation with MNP@SR and TMNP@SR (biological replicates, Data are presented as the mean ± SD, n = 3). (C) Quantification of LPS removal by MNP@SR and TMNP@SR (40 μg/mL) (biological replicates, Data are presented as the mean ± SD, n = 3). (D) Quantification of LPS removal with a fixed concentration (40 μg/mL) after incubation with MNP@SR and TMNP@SR of varied concentrations. Quantification of the decoy efficiency of MNP@SR and TMNP@SR for proinflammatory cytokines, including (E) TNF-α, (F) IFN-γ, (G) IL-1β and (H) IL-6 (biological replicates, Data are presented as the mean ± SD, n = 3). MNP@SR, macrophage-like SiO_2_-RAPA nanoparticles; TMNP@SR, TrkA overexpressed macrophage-like SiO_2_-RAPA nanoparticles.


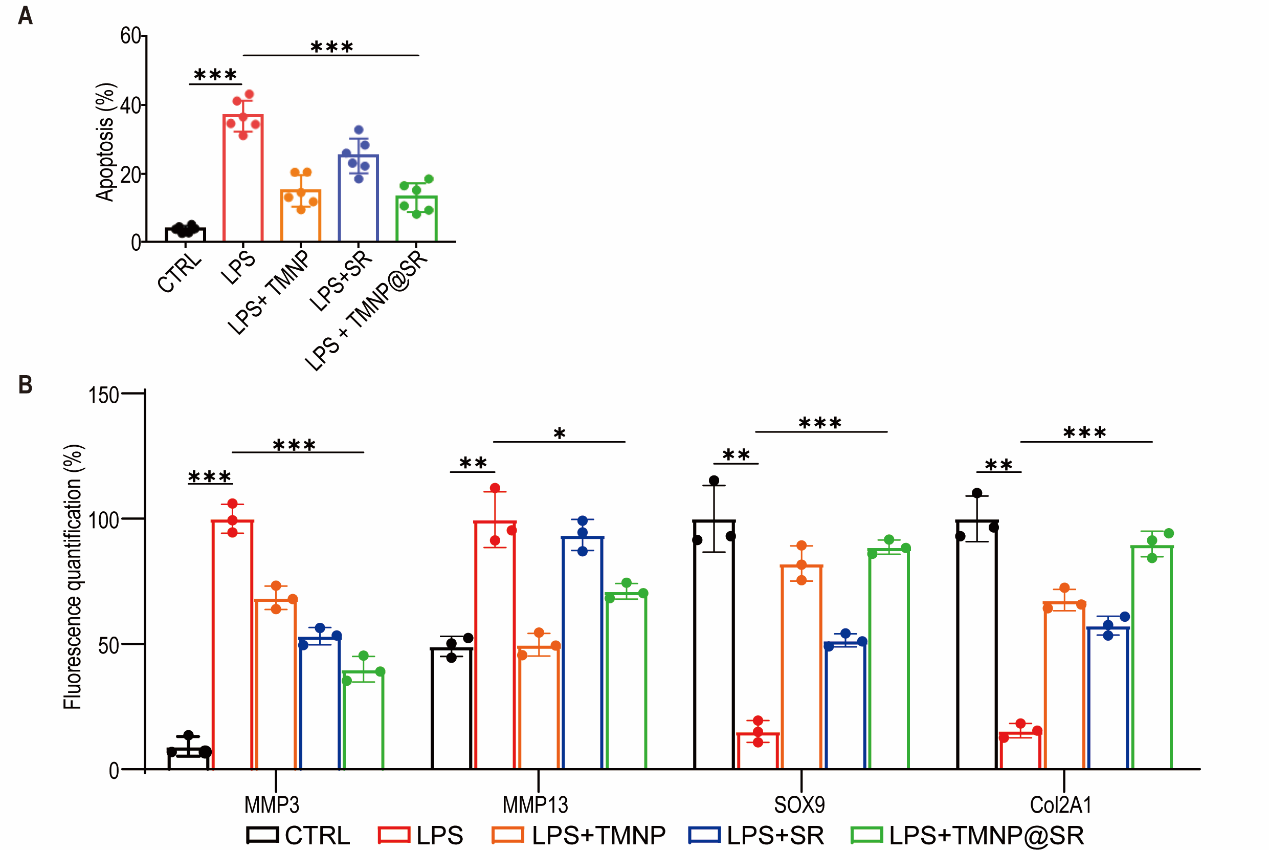


**Figure S4.** Statistical analysis of NPCs apoptosis using flow cytometry and statistical analysis of NPCs protein content. (A) The apoptosis rate was calculated as the sum of the proportion of Annexin V+ PI− and Annexin V+ PI+ cells (biological replicates, Data are presented as the mean ± SD, n = 6). (B) Quantification of immunofluorescence staining of matrix-degrading proteases (MMP3 and MMP13) and matrix anabolic factors (Col2A1, and Sox9) in NPCs with M1 polarized macrophages treated with TMNP, SR, or TMNP@SR (biological replicates, Data are presented as the mean ± SD, n = 3). ns, non-significant, *p < 0.05, **p < 0.01, ***p < 0.001. SR, mesoporous silica nanoparticles loaded with rapamycin; TMNP, TrkA overexpressed macrophage-like nanoparticles; TMNP@SR, TrkA overexpressed macrophage-like SiO_2_-RAPA nanoparticles.


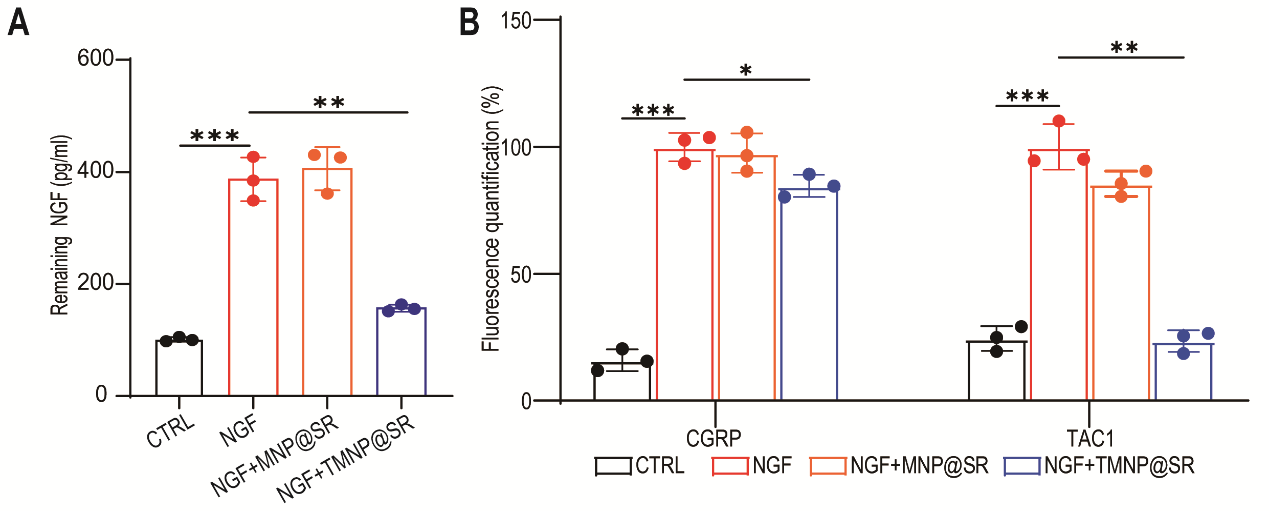


**Figure S5.** The residual NGF content in DRG supernatant and the statistical analysis of pain-related phenotype immunofluorescence. (A) Detected the concentration of inflammatory factors in dorsal root ganglion cells treated with NGF, NGF+MNP@SR, and NGF+TMNP@SR (biological replicates, Data are presented as the mean ± SD, n = 3). (B) Statistical analysis of immunofluorescence staining results of CGRP and TAC1 (biological replicates, Data are presented as the mean ± SD, n = 3). ns, non-significant, *p < 0.05, **p < 0.01, ***p < 0.001. MNP@SR, macrophage-like SiO_2_-RAPA nanoparticles; TMNP@SR, TrkA overexpressed macrophage-like SiO_2_-RAPA nanoparticles.


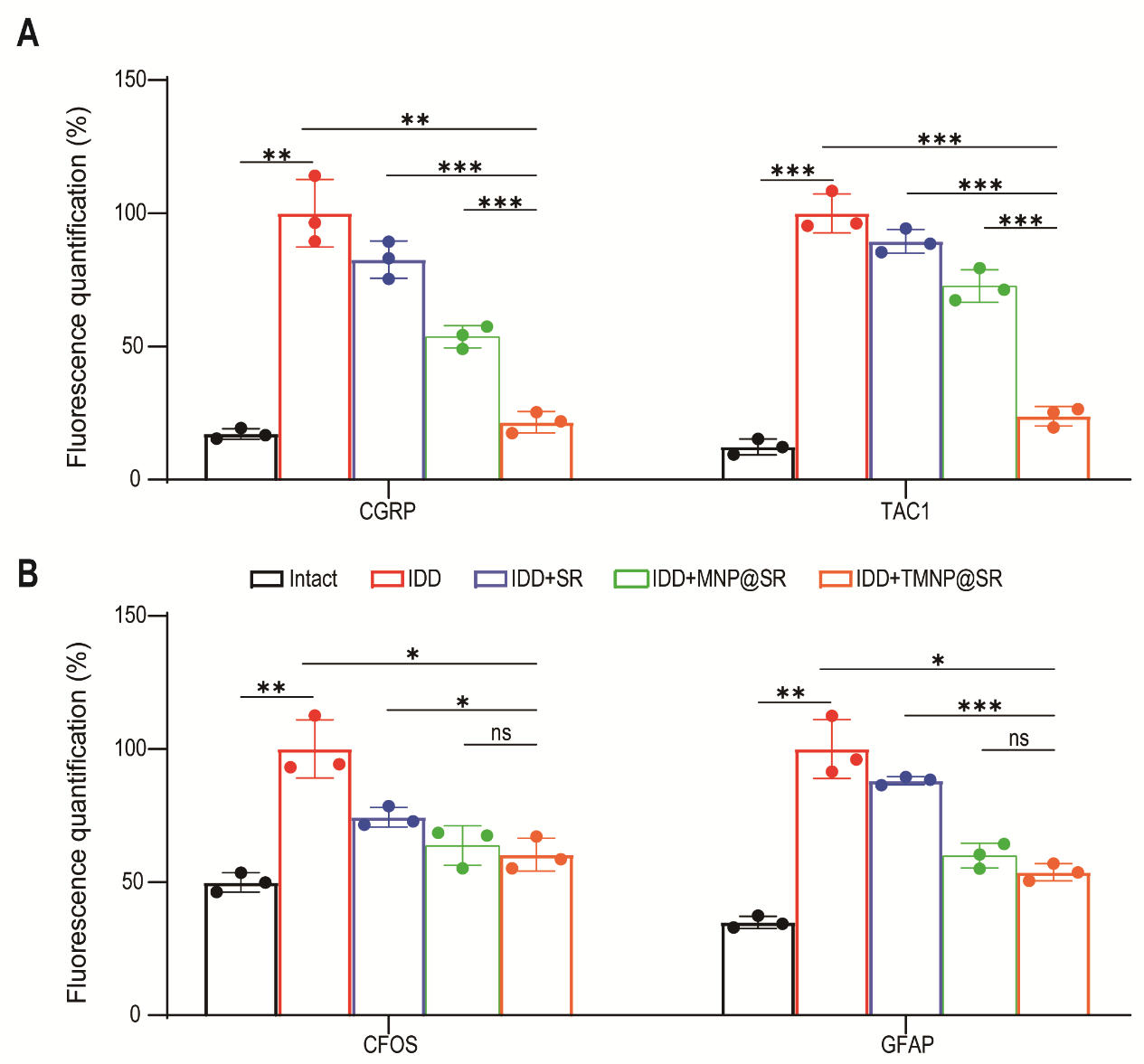


**Figure S6.** Statistical analysis of immunofluorescence in DRGs and spinal cord. (A)Statistical analysis of immunofluorescence staining results of CGRP and TAC1 (biological replicates, Data are presented as the mean ± SD, n = 3). (B)Statistical analysis of immunofluorescence staining results of CFOS and GFAP (biological replicates, Data are presented as the mean ± SD, n = 3). ns, non-significant, *p < 0.05, **p < 0.01, ***p < 0.001. IDD, Intervertebral disc degeneration; SR, mesoporous silica nanoparticles loaded with rapamycin; MNP@SR, macrophage-like SiO_2_-RAPA nanoparticles; TMNP@SR, TrkA overexpressed macrophage-like SiO_2_-RAPA nanoparticles.


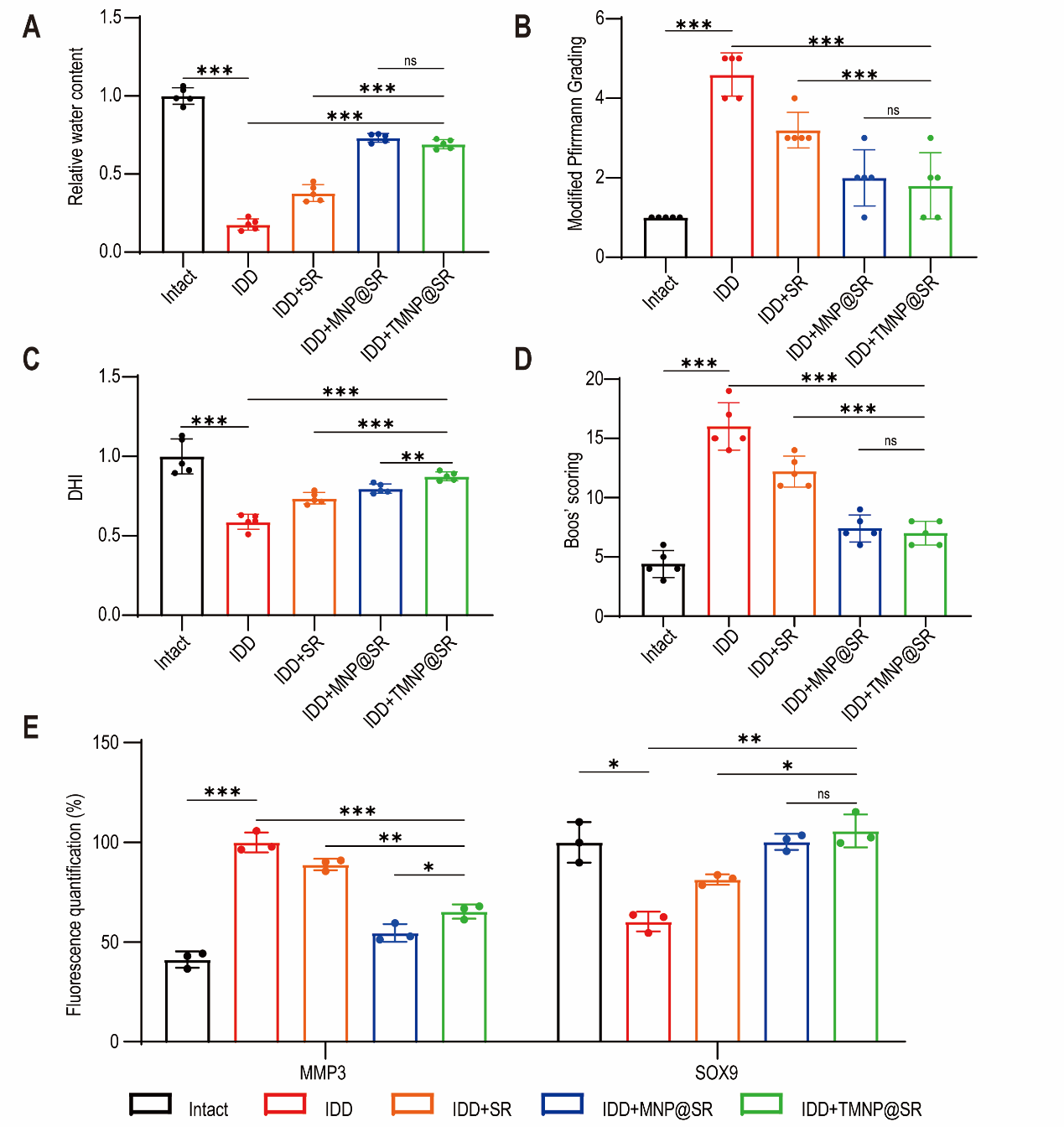


**Figure S7.** Statistical analysis of magnetic resonance imaging (MRI), X-rays, and immunofluorescence in rat intervertebral discs was performed. (A) Statistical analysis of relative water content (biological replicates, Data are presented as the mean ± SD, n = 5). (B) Statistical analysis of modified Pfirrmann grade (biological replicates, Data are presented as the mean ± SD, n = 5). (C) Statistical analysis of Representative X-ray images of disc height index (DHI) (biological replicates, Data are presented as the mean ± SD, n = 5). (D) Statistical analysis of histological score (biological replicates, Data are presented as the mean ± SD, n = 3). (E) Statistical analysis of immunofluorescence staining results of MMP3 and SOX9 (biological replicates, Data are presented as the mean ± SD, n = 3). ns, non-significant, *p < 0.05, **p < 0.01, ***p < 0.001. IDD, intervertebral disc degeneration; SR, mesoporous silica nanoparticles loaded with rapamycin; MNP@SR, macrophage-like SiO_2_-RAPA nanoparticles; TMNP@SR, TrkA overexpressed macrophage-like SiO_2_-RAPA nanoparticles.


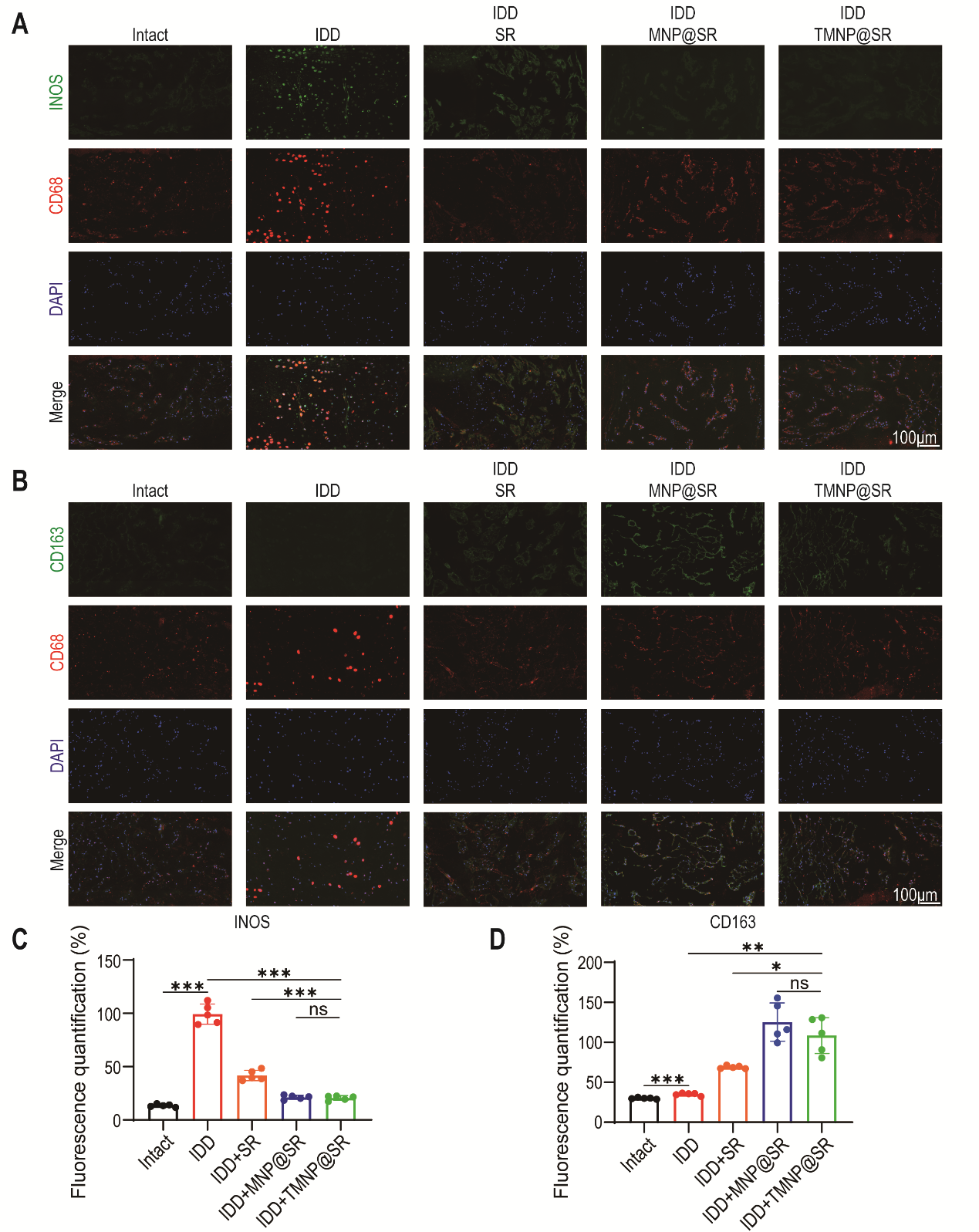


**Figure S8.** TMNP@SR modulated the macrophage polarization in rat disc tissues. Representative immunofluorescence images of iNOS (A) and CD163 (B) of rat degenerative disc samples treated with SR, MMP@SR, or TMNP@SR. Scale bars: 100 μm. Statistical analysis of the fluorescence intensity of iNOS (C) and CD163 (D) (biological replicates, biological replicates, Data are presented as the mean ± SD, n = 5). ns, non-significant, *p < 0.05, **p < 0.01, ***p < 0.001. IDD, intervertebral disc degeneration; SR, mesoporous silica nanoparticles loaded with rapamycin; MNP@SR, macrophage-like SiO_2_-RAPA nanoparticles; TMNP@SR, TrkA overexpressed macrophage-like SiO_2_-RAPA nanoparticles.
